# Evolution of the sugar receptors in insects

**DOI:** 10.1186/1471-2148-9-41

**Published:** 2009-02-18

**Authors:** Lauren B Kent, Hugh M Robertson

**Affiliations:** 1 Department of Entomology, University of Illinois at Urbana-Champaign, Urbana, IL 61801, USA

## Abstract

**Background:**

Perception of sugars is an invaluable ability for insects which often derive quickly accessible energy from these molecules. A distinctive subfamily of eight proteins within the gustatory receptor (Gr) family has been identified as sugar receptors (SRs) in *Drosophila melanogaster *(Gr5a, Gr61a, and Gr64a-f). We examined the evolution of these SRs within the 12 available Drosophila genome sequences, as well as three mosquito, two moth, and beetle, bee, and wasp genome sequences.

**Results:**

While most Drosophila species retain all eight genes, we find that the three Drosophila subgenus species have lost Gr64d, while *D. grimshawi *and the *D. pseudoobscura/persimilis *sibling species have also lost Gr5a function. The entire Gr64 gene complex was also duplicated in the *D. grimshawi *lineage, but only one potentially functional copy of each gene has been retained. The numbers of SRs range from two in the hymenopterans *Apis mellifera *and *Nasonia vitripennis *to 16 in the beetle *Tribolium castaneum*. An unusual aspect is the evolution of a novel exon from intronic sequence in an expanded set of four SRs in *Bombyx mori *(BmGr5-8), which appears to be the first example of such exonization in insects. Twelve intron gains and 63 losses are inferred within the SR family.

**Conclusion:**

Examination of the SRs in these fly, mosquito, moth, beetle, and hymenopteran genome sequences reveals that they appear to have originated independently from single ancestral genes within the dipteran and coleopteran lineages, and two genes in the lepidopteran and hymenopteran lineages. The origin of the insect SRs will eventually be illuminated by additional basal insect and arthropod genome sequences.

## Background

Sugars serve as some of the simplest, most easily metabolized forms of energy available to life. For example, despite an anautogenous female mosquito's need for a bloodmeal to nourish her developing eggs, it is the simple nectar of plants that fuels her flight muscles and daily energy needs. As sugar is a valuable resource, it seems fitting that most animals have the ability to taste sugars, and in many it forms a primary stimulatory signal for feeding. The molecular basis for sugar detection in insects has been revealed in *Drosophila melanogaster *where it involves a series of at least eight genes in the gustatory receptor (Gr) family [1-3]. The first of these is Gr5a on the X chromosome, although identification of this gene as encoding a trehalose receptor was initially confused with the neighboring *Tre *locus [4]. In phylogenetic analyses, Gr5a clusters with seven other genes on the third chromosome, including the singleton Gr61a and Gr64a-f: six genes in a tandem array [5], making all of these candidate sugar receptors (SRs). Recent work with these SRs has started to unravel their involvement in sugar detection, although much work remains to understand how these flies perceive sugars. Thorne et al. [6] and Wang et al. [7] showed that Gr5a is expressed widely in sensory neurons that detect sugars. Subsequently, Jiao et al. [8] showed that Gr5a-expressing cells also express undefined combinations of the other seven genes, and showed that Gr64a is required for sensing several sugars other than trehalose. Dahanukar et al. [9] showed that Gr61a and Gr64f are co-expressed with Gr5a in some but not all sugar-sensitive neurons, indicating that there is a complicated pattern of co-expression of these eight genes. Furthermore, they generated double-mutant flies for both Gr5a and Gr64a that cannot taste any sugars, suggesting that these two receptors co-function with the other six to achieve detection of sugars. Meanwhile, Slone et al. [10] generated a deletion mutant removing Gr64a-f and found that these flies could not detect most sugars, including trehalose, which is supposed to be detected by Gr5a. Together the evidence from these studies affirms that these eight proteins constitute the SRs in flies, and strongly suggests that they function as heterodimers, perhaps with Gr5a and Gr64a pairing with each other and/or the other less widely-expressed Gr61a and Gr64b-f. Many issues remain unresolved, including the exact ligand specificities of each heterodimeric pair of these eight SRs. Here we contribute to our understanding of these fly SRs by examining their evolution in the 11 newly available Drosophila species genomes [11], as well as more distant comparisons with the three available mosquito genomes, and the available moth, beetle, bee, and wasp genomes. This analysis reveals an unexpected history of expansion of these gene subfamilies from only one or two genes in each insect order, as well as such unusual features as evolution of a novel exon in a lineage of moth SRs.

## Methods

Homologs of the eight SR genes in *D. melanogaster *were identified in the 11 newly available Drosophila species genome sequences using the assemblies available in FlyBase as of October 2007, which are those employed in the genome paper [11], except that the *D. simulans *assembly is the "merged" assembly of six different strains. TBLASTN searches were employed to identify these genes, and gene models were constructed using the DmGrs as templates in the text editor of PAUP* v4 [12]. The *D. simulans *assembly available at FlyBase has numerous problems, including unexplained single base indels relative to the raw traces available in the Trace Archive at the National Center for Biotechnology Information (NCBI). Such errors were present in most of the genes and were corrected.

The mosquito *Anopheles gambiae *and *Aedes aegypti *gene models are from Hill et al. [13] and Kent et al. [14], but updated in light of gene models constructed for *Culex pipiens *using the CpipJ1 assembly available at VectorBase, the NCBI, the Broad Institute, and the J. Craig Venter Institute (JCVI). The *Bombyx mori *moth gene models are from Wanner and Robertson [15], while those for the red flour beetle *Tribolium castaneum *were constructed by HMR for the main genome publication [16]. The two honey bee *Apis mellifera *SRs are from Robertson and Wanner [17] and their homologs in the parasitoid wasp *Nasonia vitripennis *were built from the v1.0 assembly available from the Human Genome Sequencing Center at the Baylor College of Medicine and NCBI. The complete set of SRs is provided in a supplementary online FASTA file (Additional file 1).

All proteins were aligned using the multiple alignment program CLUSTALX with default settings [18]. The alignments were used to detect potential problems with the gene models, which were then refined. Phylogenetic analysis was performed using corrected distances, as well as supporting maximum parsimony and maximum likelihood analysis, as described in Robertson et al. [5], Robertson and Wanner [17] and Kent et al. [14]. Intron locations and phases were mapped to the protein alignment manually in the PAUP text editor and then mapped to branches in the phylogenetic tree using Dollo parsimony assuming that intron gains are unique but losses are independent events.

## Results

### The Drosophila SRs

**Figure 1:**
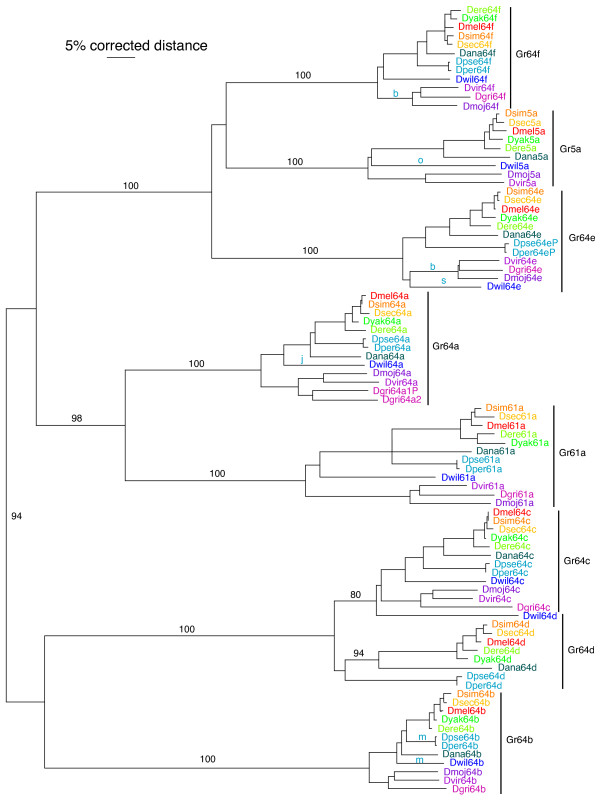
**Phylogenetic relationships of eight SRs in 12 Drosophila species**. This is a corrected distance tree based on aligned amino acids and rooted at the midpoint. The variable length and sequence N-termini upstream of the conserved "pre-peak" region (Figures 7, 8, 9, 10, 11, 12 and 13) were removed from the alignment. Species names are abbreviated to the genus plus the first three letters of the species name, and are color-coded. The eight proteins are indicated on the right. Bootstrap support from 1000 replications of uncorrected distance analysis is shown for the major branches. Most orthologous relationships are in accordance with the known species relationships, or near enough, and bootstrap support is not shown for them. Intron losses are shown in lower case light blue letters above the branches to which they map, but only if not shown in Figure 3.

**Figure 2:**
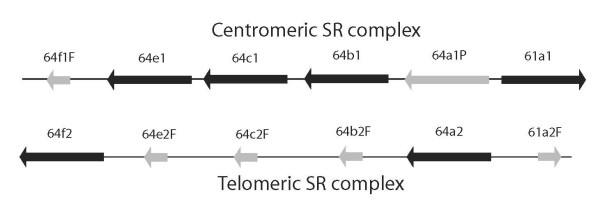
**Schematic diagram of the two SR gene complexes in *D. grimshawi***. The two complexes and their genes are labeled 1 and 2 for the centromeric and telomeric complexes, with intact genes shown as solid boxes, pseudogenes as grey boxes, and pseudogenic gene fragments as short grey boxes. Direction of transcription is shown by arrow heads. Paralogy is indicated by dashed lines. Not to scale.

For the most part the 12 available Drosophila genome sequences contain single intact orthologs for each of the eight Drosophila SR lineages (Figure 1). The only previously known exception is that Gr5a is missing from *D. pseudoobscura *[9,19] and, not surprisingly, its sibling species *D. persimilis*. There are, however, several other instances of gene subfamily evolution within this fly genus. Gr64e appears to be a pseudogene in both of these species because the intron donor splice site on the penultimate intron starts with GA instead of the canonical GT. Gr5a is a severely damaged pseudogene in the Hawaiian *D. grimshawi*, and is not included in the tree analysis. In addition, there was a duplication of the entire 3^rd ^chromosome gene complex in *D. grimshawi*, roughly 2.6 Mbp apart, followed by the loss or pseudogenization of each gene in one or the other version of the complex, leaving a single intact copy of each gene (Figure 2). Thus the centromeric complex retains a functional copy of Gr61a, a pseudogenic copy of Gr64a, and functional copies of Gr64b, Gr64c, and Gr64e, followed by a fragment of Gr64f, while the telomeric complex has an intact copy of Gr64a and Gr64f, and fragments of Gr61a, Gr64b, Gr64c, and Gr64e. We designate genes in the centromeric complex by the number "1" after their name and the telomeric complex by the number "2". In addition, Gr64d is missing from the three Drosophila subgenus species, *D. virilis*, *D. mojavensis*, and *D. grimshawi*, so this loss predates the duplication of the complex in the *D. grimshawi *lineage. Judging from the branch lengths of the DgriGr64a1P/2 copies, this gene complex duplication is relatively old and may be present in all Hawaiian Drosophila. We have not determined how extensive the duplication is, but it presumably involves multiple flanking genes as well. Another slightly unusual problem is the phylogenetic placement of what we are calling Gr64d in *D. willistoni*. This gene is in the expected location for Gr64d, that is between Gr64c and Gr64e, however phylogenetically it is clearly closer to the Gr64c genes than the Gr64d genes. There is no simple explanation for this situation. A duplication of Gr64c in *D. willistoni *followed by loss of the original Gr64d gene should lead to our Gr64d clustering with the *D. willistoni *Gr64c, and there is no evidence of a partial gene conversion event. We are also able to date roughly the movement of Gr5a and Gr61a from the tandem complex of Gr64a-f. All Drosophila species appear to have Gr5a on their X chromosomes, so this gene relocation predates the genus. However, Gr61a is located in inverse orientation at the 5' end of the complex, in all species up to *D. ananassae*, so it must have relocated thereafter. Finally, Gr61a is relocated to the X chromosome in *D. yakuba*. The result is that the number of apparently intact SRs in these 12 Drosophila species is six in *D. pseudoobscura/persimilis *and *D. grimshawi*, seven in *D. virilis *and *D. mojavensis*, and eight in the remainder of the species.

Our analysis of the Drosophila Grs differs somewhat from that recently reported in Gardiner et al. [20], primarily in that they ignore the Gr5a pseudogene in *D. grimshawi*, and list multiple copies of Gr61a (5 genes and 3 pseudogenes), Gr64a (4/3), and Gr64b (2/1) in this species beyond what we include (Figure 2). This may be because they used early assemblies from January 2006, which for this species might have had multiple haplotypes alternatively assembled. Remnants of these remain in the October 2007 assemblies as short contigs and were not included in our analysis.

### The mosquito SRs

**Figure 3:**
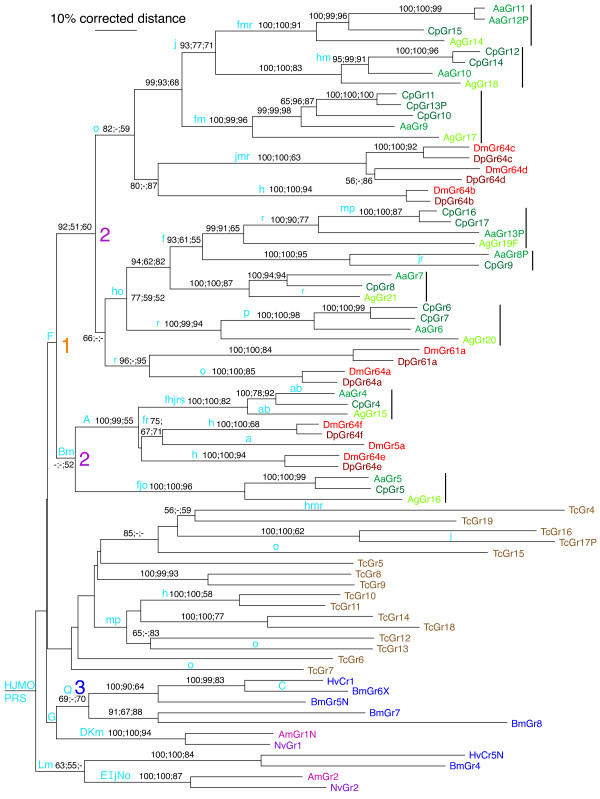
**Phylogenetic relationships of SRs in insects**. This corrected distance tree was rooted with the AmGr2/NvGr2 and HvCr5/BmGr4 lineage as the outgroup, based on their apparent basal clustering in larger analyses of the entire Gr family. Bootstrap support above 50% from 1000 replications of full heuristic uncorrected distance analysis, 1000 bootstrap replications of full heuristic maximum parsimony analysis, and 25000 maximum likelihood quartet puzzling steps are shown above relevant branches. Intron gains and losses are indicated by light blue upper case and lower case letters, respectively, above branches to which they are mapped by simple presence-absence Dollo parsimony. Drosophila SRs are shown in shades of red, mosquito green, beetle brown, moth blue, and hymenopteran purple. Only the *D. pseudoobscura *SRs are included along with those from *D. melanogaster *to help balance the dipteran SR analysis. Inferred orthologous relationships within the three mosquito species are indicated by bars on the right. The large orange 1 and purple 2's indicate the two major tandem duplications hypothesized at the base of the dipteran SR evolution (see Figure 4). The blue 3 indicates the origin of the novel exon and hence also intron "q" in the expanded moth SR lineage.

We find that *An. gambiae *and *Ae. aegypti *have eight and seven functional SRs, respectively. In addition, *Ae. aegypti *also has three pseudogenes. [13,14]. We examined the newly available *Culex pipiens *genome sequence and find that this mosquito, which is ~50 Myr diverged from *Ae. aegypti *[21,22], has 14 sugar receptor genes, of which one is a pseudogene (Figure 3). Five of these extra receptor genes are the result of relatively recent duplications within the *C. pipiens *lineage. Phylogenetic analysis reveals, however, that what we had earlier considered to be an orthologous, albeit rather divergent, relationship of AgGr19 and AaGr13P [14], is in fact a paralogous comparison, because *C. pipiens *has clear orthologs of each of these receptors. Thus *An. gambiae *has lost the ortholog of AaGr13P/CpGr16/17, while *Ae. aegypti *has lost the ortholog of AgGr19/CpGr6/7. We therefore infer that most mosquito lineages have nine SR gene lineages, although not all are present and intact in all species, and some have been duplicated in some species.

### Evolution of the fly SRs

**Figure 4:**
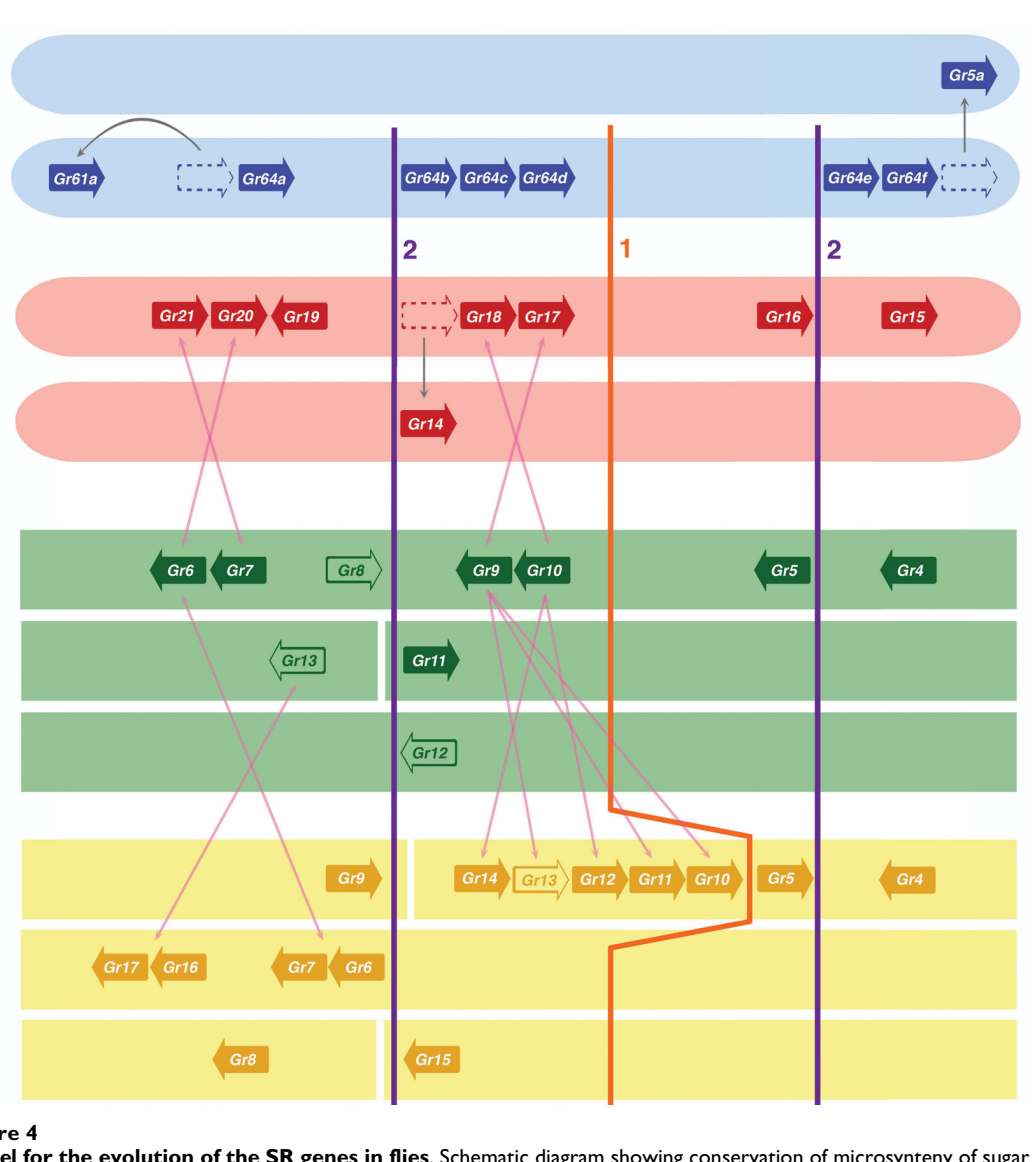
**Model for the evolution of the SR genes in flies**. Schematic diagram showing conservation of microsynteny of sugar Grs in *Drosophila melanogaster *(blue), *Anopheles gambiae *(red), *Aedes aegypti *(green) and *Culex pipiens *(yellow). Most of the genes share both phylogenetic relatedness and similar placement in chromosomes (*Ae. gambiae *and *D. melanogaster*) and supercontigs (*Ae. aegypti *and *C. pipiens*) across the four species, even after duplication events (shown with line 1 and lines 2, as in Fig. 3). Although it appears that some genes have moved within their respective genomes in *D. melanogaster *and *An. gambiae *(shown with grey arrows), their phylogenetic relationships (Fig. 3) reveal where they most likely had previously resided on the chromosomes. Likewise, it appears that there has been some level of gene shuffling in *Ae. aegypti *and *C. pipiens *(shown with pink arrows) with respect to the arrangements seen in *D. melanogaster *and *An. gambiae*. Although the genes have not yet been mapped to *Aedes *and *Culex *chromosomes, the ordered supercontigs of the genome sequences are likely accurate representations of microsynteny in each species.

As noted in Kent et al. [14], the relationships of the eight Drosophila SR lineages and the then-eight and now-nine mosquito SRs is not one of simple orthology, but rather a complex pattern of gene duplications and losses, presumably reflecting even older gene subfamily events like the more recent ones seen within Drosophila and mosquitoes. Although our phylogenetic analysis does not provide bootstrap support for the single basal root of this fly SR lineage in Figure 3, it is supported by the apparent acquisition of intron F which is unique to the flies (see details below), so we hypothesize that the lineage arose from a single ancestral SR gene in an early fly. This does not mean that an early fly had only one SR gene, because it could well have had several, with all others being lost subsequently. We hypothesize that this single gene underwent a simple tandem gene duplication (large orange "1" in Figures 3 and 4), leading to two lineages. Then before the split of the major dipteran suborders (Brachycera and Nematocera, represented by Drosophila and mosquitoes, respectively), ~260 Mya [23], each of these lineages underwent simple tandem duplications (large purple 2's in Figures 3 and 4). Subsequent to this major organismal lineage split, the four existing SR genes underwent independent duplications, losses, and transpositions, leading to the current SR phylogeny of Drosophila and mosquitoes. In particular, the drosophilid flies have lost the lineage related to AgGr16/AaGr5/CpGr5. As a consequence of this convoluted history, ligand specificity determined for the Drosophila SRs cannot be directly transferred to the mosquito SRs, although it might be suggestive.

### The Tribolium castaneum beetle SRs

The *T. castaneum *Gr family was described by HMR in the main genome paper [16], however in the phylogenetic analysis performed therein, the 16 SRs did not cluster as a single lineage, perhaps because the analysis included the entire Gr family. With the current phylogenetic analysis restricted to the SR subfamily, we believe we obtain refined clustering of the Tribolium SRs into a single lineage, however again there is no bootstrap support for this monophyly. Furthermore, we again are not proposing that beetles once had a single SR, simply that the existing Tribolium SR complement of 16 genes is monophyletic. The relatively high divergence of the Tribolium SRs from the other SRs precludes any suggestion of ligand specificity and we propose that all ligand specificity of the Tribolium SRs evolved independently within the beetle lineage.

### The moth SRs

**Figure 5:**
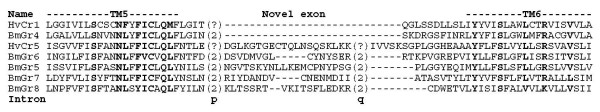
**A novel exon in BmGr5-8**. The amino acids of the TM5 and TM6 regions encoded by the ends of the exons flanking the novel exon are shown aligned with each other for the seven moth SRs. Amino acids shared by at least six of the sequences are highlighted in bold font. Like other SRs, BmGr4 does not have the novel exon, and the same is predicted for HvCr1. The location of the phase 2 intron in BmGr4 (intron p in Figure 6) is shown. BmGr5-8 have a short novel exon encoding 15–20 unconserved amino acids, and an additional flanking phase 2 intron (intron q in Figure 6). HvCr5 is predicted to have an intron q, however the precise location cannot be predicted.

Krieger et al. [24] included cDNA sequences for two SRs in their initial description of a set of candidate chemoreceptors from the noctuid moth *Heliothis virescens *based on private partial genome sequences generated by Bayer and Exelixis Corporations. They subsequently obtained cDNAs, called Cr1 and Cr5 in their publication, which turn out to represent two divergent SR lineages within moths, as revealed by examination of the Gr family in the genome of the silkmoth *Bombyx mori *[25,26]. Details of our analysis of the Gr family in *B. mori *are published elsewhere [15], but we include the five SRs here for completeness. *B. mori *has a single ortholog of HvCr5, which we have named BmGr4 (BmGr1-3 are the carbon dioxide receptors [27]). *B. mori *also has a simple ortholog of HvCr1, named BmGr6, as well as an expansion of three other genes, BmGr5, BmGr7, and BmGr8, in a monophyletic lineage with HvCr1/BmGr6. The monophyly of this lineage is supported not only by bootstrapping, but by each member's (BmGr5-8 and we predict HvCr1 as well) remarkable possession of a newly evolved short exon from within the ancestral phase 2 intron p (see intron details below). This novel exon is supported by a partial cDNA sequence for BmGr6, which was submitted to GenBank as "candidate olfactory receptor BmOR20" [GenBank:BAF31192.1 by T. Sakusai, Y. Hashimoto, and T. Nishioka in 2006], but which has a deletion of an upstream exon preventing complete translation. It has also been confirmed by sequencing of an RT/PCR product between the flanking exons for BmGr8 [15]; [GenBank:EU769119], and is inferred bioinformatically for BmGr5 and BmGr7. It encodes 15–20 amino acids in BmGr5-8 (Figure 5), and at least that number of amino acids in HvCr1 (where the genome sequence is not available, but the cDNA encodes these extra amino acids). These amino acids show no sequence conservation and are part of the extracellular loop 3 (ECL3; see below). This loop is usually very short in all insect Grs, so this evolution of a novel exon encoding a short stretch of variable amino acids that lengthen ECL3 is unusual.

### The hymenopteran SRs

We examined the newly available parasitoid jewel wasp *Nasonia vitripennis *genome sequence for SRs, and find only simple orthologs of the *Apis mellifera *Gr1 and Gr2 genes [17], sharing 64 and 55 percent sequence identity, respectively (Figure 3). Bees and wasps form a sister group within the order Hymenoptera, so it appears likely that they all have only these two SRs. These two genes are next to and facing each other in a contig of the *N. vitripennis *genome assembly, but 2 Mbp apart on chromosome 5 in *A. mellifera*, suggesting that they have remained neighbors in the wasp lineage since their duplication in a common ancestor, but became separated in the bee lineage. These hymenopteran SRs form two sister lineages with the two SR lineages in moths, suggesting that these are rather old gene lineages. Although bootstrap support for this notion is not robust, much like the fly SR lineage sharing the unique intron f, each of these SR lineages in moths and hymenopterans has a unique intron position (g and l, respectively, see below).

### Intron evolution

**Figure 6:**
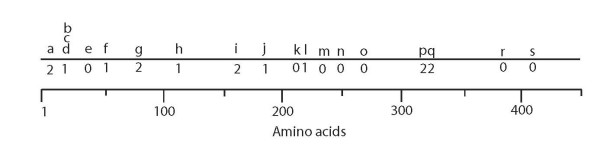
**Intron locations and phases in the insect SR genes**. Introns are named in an alphabetical series from 5' to 3' ends of the genes, positioned above a line representing a 450aa SR, with the longer N-terminus typical of the DmGr5a and 64e/f lineage. Hence the coordinates of the introns are ~20 aa later than those shown in Figure 4 of Robertson et al. [5]. Correspondence between intron names in Robertson et al. [5] and those herein is: a/a, b/b, d/f, j/h, o/j, r/m, y/o, a'/p, 2/r, 3/s. Intron phase is shown below the line (0 is between codons, 1 is after the first codon position, and 2 after the second codon position).

Robertson et al. [5] inferred considerable intron evolution within the insect chemoreceptor superfamily, and specifically the Gr family, from comparisons of the genes within *Drosophila melanogaster *alone, something that was also evident from the initial gene descriptions (e.g. [1,28]). Examination of this issue within the three-gene carbon dioxide lineage revealed considerable intron gain and loss even just within this gene lineage [27]. Intron evolution within these SR genes is again evident. We rename the introns from those in Robertson et al. [5], because otherwise the alphabetical naming system becomes cumbersome. The intron locations and phases are shown in Figure 6, and their gains and losses are mapped on the tree in Figure 3. Inclusion of the other insect SRs leads to some revision of when some introns were gained, compared with Figure 3 in Robertson et al. [5]. For example, intron f (d in [5]) can now be inferred to have been gained in the single ancestor of the fly SR genes, rather than being diagnostic of the SRs in general, because it is absent in the non-Dipteran genes. Indeed, it now provides useful support for our proposal that the fly SRs are monophyletic, all originating from a single SR gene in a basal fly lineage. Intron f was then lost independently six times within the dipteran gene expansion. Conversely, intron r (m in [5]), which was initially inferred to have been gained within the Drosophila SRs in Robertson et al. [5], is clearly much older because it is shared by the Bombyx and Tribolium SRs. Indeed, we now infer that it is ancestral for the entire SR subfamily.

We infer 12 intron gains within the SRs (excluding hjmoprs), including the two N-terminal introns (a and b) in the Drosophila Gr5a/Gr64e/Gr64f lineage and mosquito relatives, the splitting of the ancestral phase 2 intron p in the expanded moth SR lineage yielding novel intron q, and eight other novel introns in the non-dipteran genes. Most of these intron locations are not only unique within this SR lineage, but also within the entire Gr family, which represents most of the diversity of the insect chemoreceptor superfamily [5]. Indeed, only the two C-terminal introns r and s (2 and 3 in [5]) are shared across the Gr family. Intron losses are rather more frequent, totaling 58 in Figure 3, plus seven in the Drosophila species in Figure 1 that do not overlap with Figure 3, for a total of 65. The two C-terminal and ancient introns, r and s, reveal the extremes of intron loss, with intron r being lost 10 times while intron s was only lost twice, in a mosquito lineage and in DwilGr64e. It remains unclear why the final intron is so seldom lost. This pattern is found not only in the SRs, but is consistent throughout the entire superfamily, as this C-terminal intron is almost always present. The only major ambiguity in this analysis of intron evolution is the series of phase 1 introns near the N-terminus, named b, c, and d. These three introns are in roughly the same location, but are found in subsets of the dipteran, moth, and hymenopteran SRs. Unfortunately, the N-terminal sequences are so divergent across these three insect orders that they cannot confidently be considered to be homologous intron placements, and given their disparate locations in the tree, are considered here to be independent gains. As was true for the carbon dioxide receptors [27], and appears generally true across entire genomes (e.g. [29]), intron losses are more frequent in the Diptera, with all dipteran gene lineages having lost at least one intron, while some moth, beetle, and hymenopteran lineages have lost none and some only gained introns (although it is also formally possible that some of these non-dipteran introns are ancestral to the SR family and were lost from the dipteran gene lineages).

### Distinctive features of SRs

**Figure 7:**
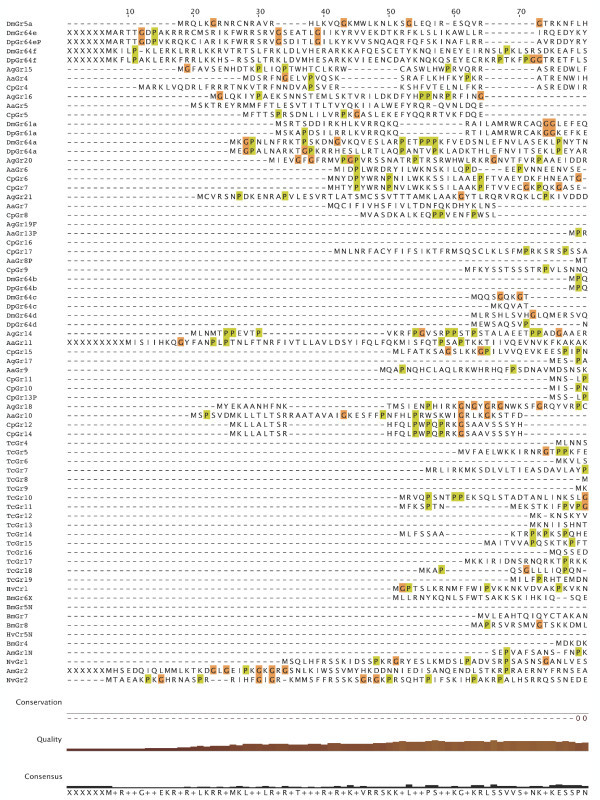
**Multiple alignment of the insect SRs**. This is the CLUSTALX alignment employed for the phylogenetic analysis in Figure 3, except that positions of uncertain alignment and large gaps, specifically alignment positions 1–82, 150–160, 209–219, 340–355, 405–435, and 531–559, were excluded from the phylogenetic analysis. The Xs at the start of some sequences were added to facilitate splitting the figure into six convenient parts. Alignment positions are shown at the bottom, along with the "conservation" histogram from CLUSTALX. The predicted TM domains are evident as vertical bands of hydrophobic amino acids shaded blue, and the "pre-peak" is approximately alignment positions 84–103, TM1 is 121–142, TM2 is 161–189, TM3 is 224–234, TM4 is 291–314, TM5 is 378–398, TM6 is 438–457, and TM7 is 511–529.

**Figure 8:**
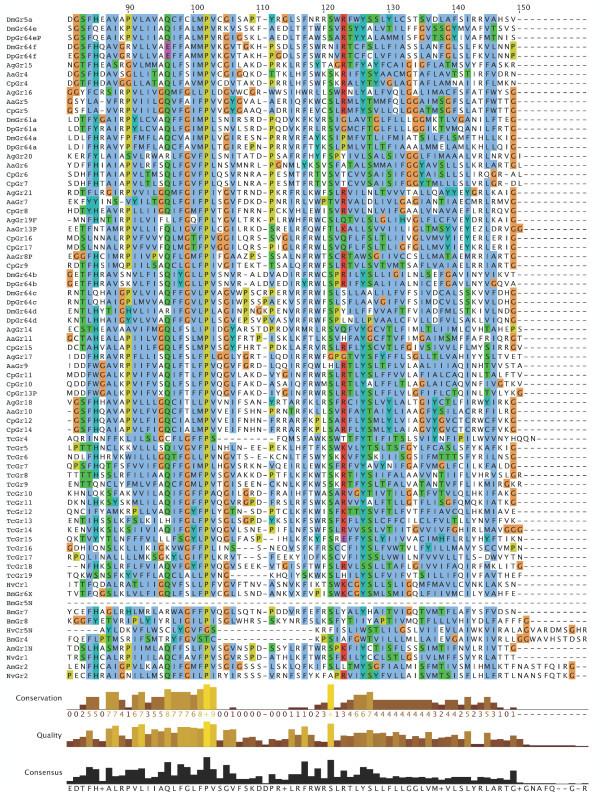
**Multiple alignment of the insect SRs**. This is the CLUSTALX alignment employed for the phylogenetic analysis in Figure 3, except that positions of uncertain alignment and large gaps, specifically alignment positions 1–82, 150–160, 209–219, 340–355, 405–435, and 531–559, were excluded from the phylogenetic analysis. The Xs at the start of some sequences were added to facilitate splitting the figure into six convenient parts. Alignment positions are shown at the bottom, along with the "conservation" histogram from CLUSTALX. The predicted TM domains are evident as vertical bands of hydrophobic amino acids shaded blue, and the "pre-peak" is approximately alignment positions 84–103, TM1 is 121–142, TM2 is 161–189, TM3 is 224–234, TM4 is 291–314, TM5 is 378–398, TM6 is 438–457, and TM7 is 511–529.

**Figure 9:**
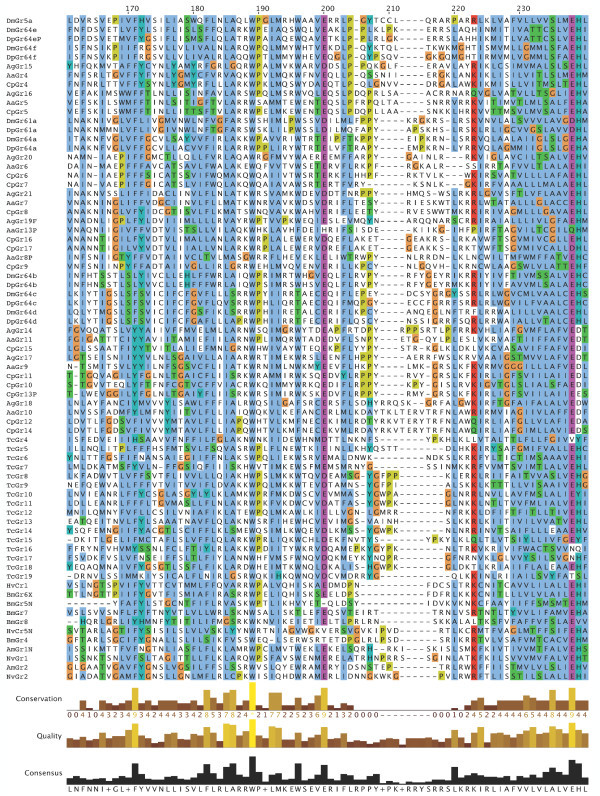
**Multiple alignment of the insect SRs**. This is the CLUSTALX alignment employed for the phylogenetic analysis in Figure 3, except that positions of uncertain alignment and large gaps, specifically alignment positions 1–82, 150–160, 209–219, 340–355, 405–435, and 531–559, were excluded from the phylogenetic analysis. The Xs at the start of some sequences were added to facilitate splitting the figure into six convenient parts. Alignment positions are shown at the bottom, along with the "conservation" histogram from CLUSTALX. The predicted TM domains are evident as vertical bands of hydrophobic amino acids shaded blue, and the "pre-peak" is approximately alignment positions 84–103, TM1 is 121–142, TM2 is 161–189, TM3 is 224–234, TM4 is 291–314, TM5 is 378–398, TM6 is 438–457, and TM7 is 511–529.

**Figure 10:**
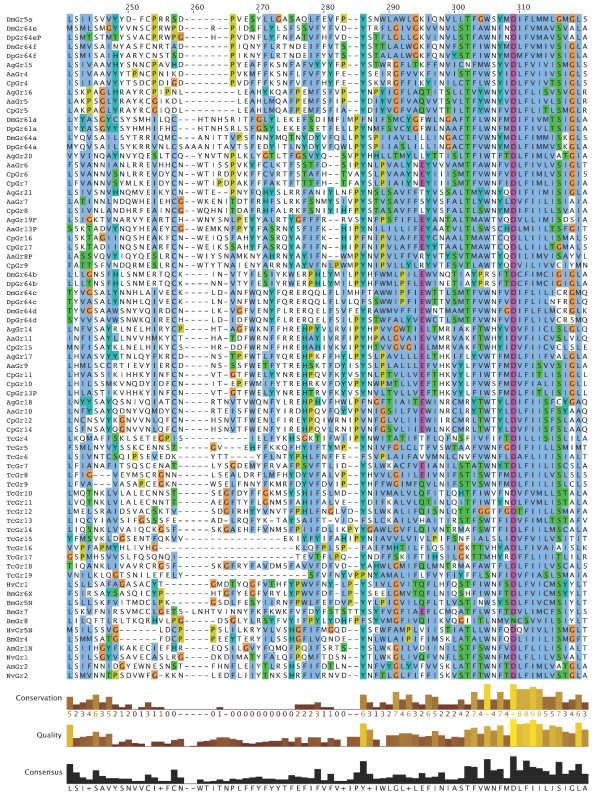
**Multiple alignment of the insect SRs**. This is the CLUSTALX alignment employed for the phylogenetic analysis in Figure 3, except that positions of uncertain alignment and large gaps, specifically alignment positions 1–82, 150–160, 209–219, 340–355, 405–435, and 531–559, were excluded from the phylogenetic analysis. The Xs at the start of some sequences were added to facilitate splitting the figure into six convenient parts. Alignment positions are shown at the bottom, along with the "conservation" histogram from CLUSTALX. The predicted TM domains are evident as vertical bands of hydrophobic amino acids shaded blue, and the "pre-peak" is approximately alignment positions 84–103, TM1 is 121–142, TM2 is 161–189, TM3 is 224–234, TM4 is 291–314, TM5 is 378–398, TM6 is 438–457, and TM7 is 511–529.

**Figure 11:**
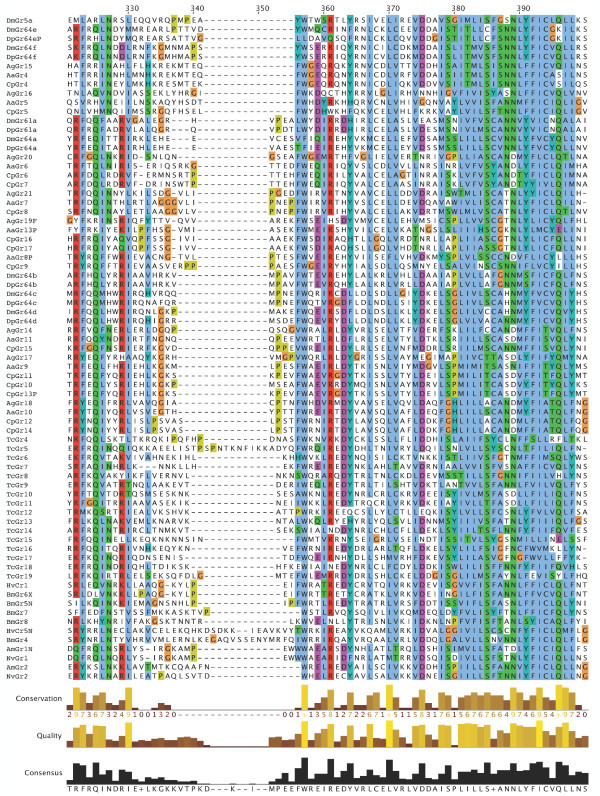
**Multiple alignment of the insect SRs**. This is the CLUSTALX alignment employed for the phylogenetic analysis in Figure 3, except that positions of uncertain alignment and large gaps, specifically alignment positions 1–82, 150–160, 209–219, 340–355, 405–435, and 531–559, were excluded from the phylogenetic analysis. The Xs at the start of some sequences were added to facilitate splitting the figure into six convenient parts. Alignment positions are shown at the bottom, along with the "conservation" histogram from CLUSTALX. The predicted TM domains are evident as vertical bands of hydrophobic amino acids shaded blue, and the "pre-peak" is approximately alignment positions 84–103, TM1 is 121–142, TM2 is 161–189, TM3 is 224–234, TM4 is 291–314, TM5 is 378–398, TM6 is 438–457, and TM7 is 511–529.

**Figure 12:**
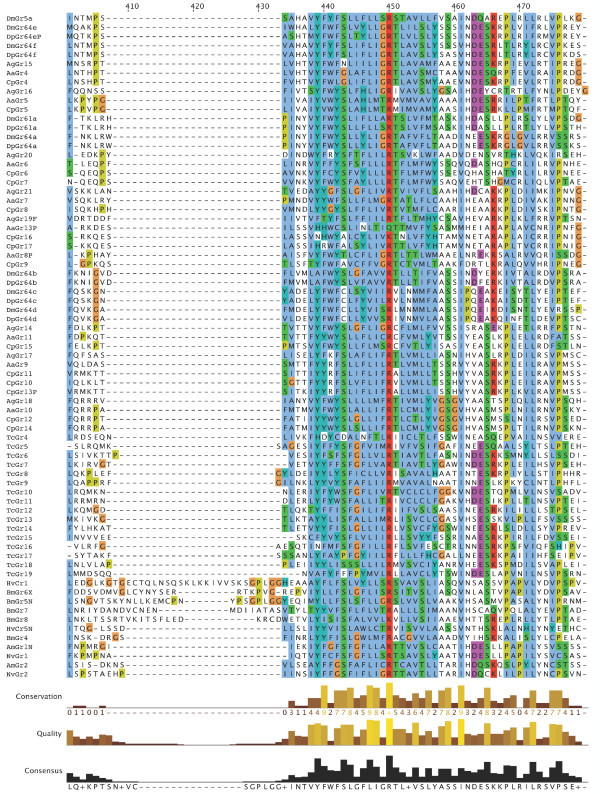
**Multiple alignment of the insect SRs**. This is the CLUSTALX alignment employed for the phylogenetic analysis in Figure 3, except that positions of uncertain alignment and large gaps, specifically alignment positions 1–82, 150–160, 209–219, 340–355, 405–435, and 531–559, were excluded from the phylogenetic analysis. The Xs at the start of some sequences were added to facilitate splitting the figure into six convenient parts. Alignment positions are shown at the bottom, along with the "conservation" histogram from CLUSTALX. The predicted TM domains are evident as vertical bands of hydrophobic amino acids shaded blue, and the "pre-peak" is approximately alignment positions 84–103, TM1 is 121–142, TM2 is 161–189, TM3 is 224–234, TM4 is 291–314, TM5 is 378–398, TM6 is 438–457, and TM7 is 511–529.

**Figure 13:**
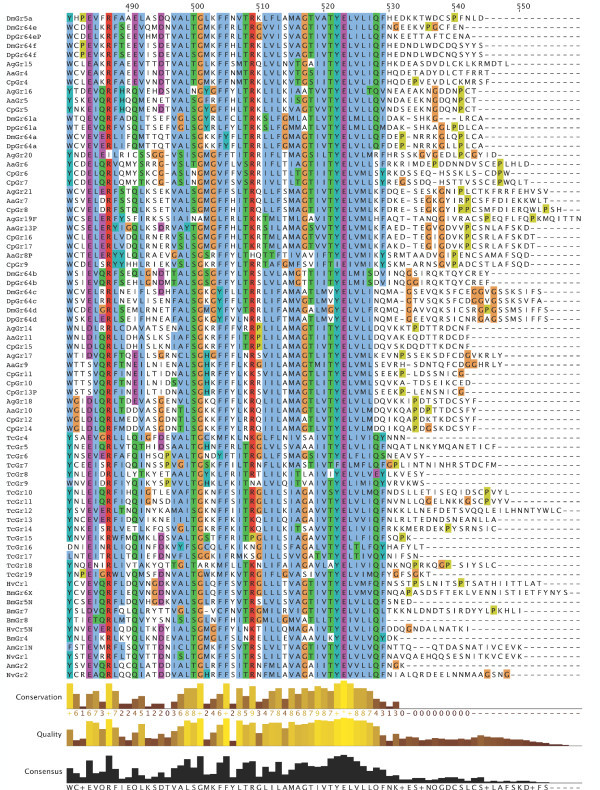
**Multiple alignment of the insect SRs**. This is the CLUSTALX alignment employed for the phylogenetic analysis in Figure 3, except that positions of uncertain alignment and large gaps, specifically alignment positions 1–82, 150–160, 209–219, 340–355, 405–435, and 531–559, were excluded from the phylogenetic analysis. The Xs at the start of some sequences were added to facilitate splitting the figure into six convenient parts. Alignment positions are shown at the bottom, along with the "conservation" histogram from CLUSTALX. The predicted TM domains are evident as vertical bands of hydrophobic amino acids shaded blue, and the "pre-peak" is approximately alignment positions 84–103, TM1 is 121–142, TM2 is 161–189, TM3 is 224–234, TM4 is 291–314, TM5 is 378–398, TM6 is 438–457, and TM7 is 511–529.

The SRs form a distinctive subfamily, with a long branch connecting them to the rest of the Gr family [5]. They appear, therefore, to have been evolving independently from the rest of the family for some time, which in part explains their distinctive set of introns. SRs are also slightly longer than most Grs, most often attributed to an N-terminal extension. This extension is particularly long in the DmGr5a/DmGr64e/DmGr64f and related mosquito SR lineage, which includes one or two additional N-terminal exons (separated by introns a and/or b in Figure 6, except for AaGr4 and AgGr15 which lost both introns). All SRs have a "pre-peak" of hydrophobic amino acids in the N-terminus that is sometimes predicted to be an eighth TM domain by various TM-domain prediction programs (see below). The amino acids within this "pre-peak" region are remarkably well conserved, with a motif that can be described as hHxAh(G/A/S)Phhhh(G/A/S)Qhh(G/A/S)hhPh, where h stands for any hydrophobic amino acid (F, I, L, M, or V; alignment positions 84–103 in Figures 7, 8, 9, 10, 11, 12 and 13). The final proline is the most conserved position. This motif is shared by most other Grs, including the carbon dioxide receptors, which in larger phylogenetic analyses usually form the sister group to the SRs. However, the somewhat conserved histidine at the start of the motif is distinctive to the SRs. The otherwise unconserved TM1 domain starts with a well-conserved serine (S alignment position 121) that is only shared with the carbon dioxide receptors, while the TM2 domain ends with a completely conserved tryptophan (W 189) that is idiosyncratically present in other Grs. TM3 has a highly conserved glutamic acid (E 238), while TM4 has a completely conserved aspartic acid (D 309), both seemingly unique to the SRs, although these regions are poorly aligned across all Grs. The intracellular loop 2 (ICL2) contains a highly conserved tryptophan (W 357), shared only with the carbon dioxide receptors, followed after four amino acids by a distinctively conserved arginine (R 361). The C-terminal TM6/ICL3/TM7 region is the most highly conserved region of the Grs, so it is not surprising that the SRs contain several conserved residues here that are shared with the rest of the Gr family, including the TY (521/2) and QF (528/9) pairs in TM7, although the QF pair was replaced in about half the fly SRs. The most distinctive residue in the SRs is a completely conserved glutamic acid (E 523) immediately after the TY pair in TM7. In other Grs this residue is usually a hydrophobic amino acid. This glutamic acid is seen in no other available insect Grs, hence is apparently diagnostic for the SR subfamily. Its conservation within the SR subfamily suggests that it somehow plays a crucial role in the perception of sugars.

### Membrane topology

**Figure 14:**
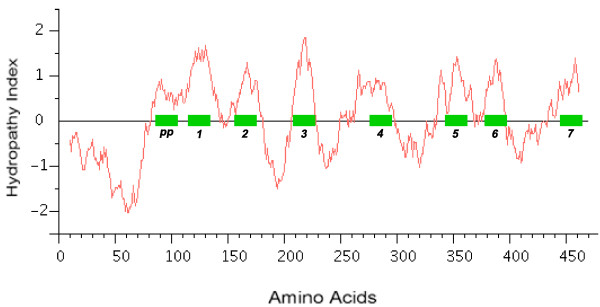
**Hydropathy plot and predicted TM domains for AmGr2**. Regions of hydrophobic amino acids yield stretches of positive hydropathy, and these are predicted to be TM domains by the ConPredII prediction program (indicated by thick green bars).

**Figure 15:**
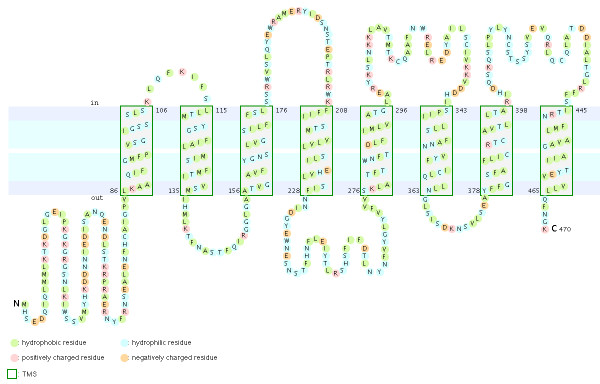
**Predicted membrane topology for AmGr2**. ConPredII predicts eight TM domains for this protein and many other SRs, usually as here with the N-terminus internal and the C-terminus external.

Recent studies have shown that the insect Ors, while most likely containing seven TM domains, have the opposite membrane topology to that of the G-protein coupled receptors that constitute chemoreceptors in vertebrates and nematodes [30-33]. That is, their N-termini are internal while their C-termini are external. We believe this topology is present throughout the Gr family as well and hence the entire superfamily [27]. These SRs provide a particularly clear illustration of this topology. Examination of the CLUSTALX alignment in Figure 7 shows clearly the relatively long intracellular (IC) loop 2 between TM4 and TM5, and IC3 between TM6 and TM7. Furthermore, these intracellular loops each have several conserved positively charged arginine (R) and lysine (K) residues, in agreement with the "positive inside" rule of von Heijne [34,35]. In contrast, the extracellular loop 3 between TM5 and TM6 is particularly short (except in the lepidopteran lineage with a novel exon), and devoid of conserved positively charged residues. Similar trends apply to the more N-terminal loops. A remaining uncertainty with respect to the membrane topology of these SRs, and Grs in general, is the presence of a "pre-peak" of hydrophobic amino acids that is less evident in the Ors, the only family studied experimentally to date. This pre-peak is around 21 amino acids long, the minimum required for a TM domain. For most of the SRs, it is predicted to be a TM domain by most hydropathy and TM domain prediction programs, as summarized in the ConPredII website [36]. Most of these proteins are predicted to have eight TM domains with the N-terminus external, although the range is from six to nine TM domains (insect chemoreceptor TM domains are seldom as well-defined as those of most other TM proteins, while TM4 is sometimes split into two). An example of these hydropathy plots, predicted TM domains, and predicted topology is shown for AmGr2 in Figures 14 and 15. Further experimental work will be required to resolve this issue, however, it has now been shown that at least the Ors are in fact ligand-gated ion channels [33,37,38], and the same is surely true of these Grs.

## Discussion

Our analysis of the evolution of the SRs in insects reveals a remarkable pattern (Figure 3). Each major lineage of SRs within an insect order appears to have originated from just one or two genes. Thus we hypothesize that all the fly SRs, all the Tribolium SRs, and most of the moth SRs originated from a single basal gene within each organismal lineage. In contrast, both moths and the hymenopteran wasp/bee lineage appear to have shared two SRs lineages for a long time. Recent work on the SRs in *D. melanogaster *strongly suggests that, like the Ors and the carbon dioxide receptors, they function as heterodimers [9,10,39]. If this is the case, then we can predict that the two existing SRs in the wasp/bee lineage function as a single receptor capable of recognizing all sugars that these hymenopterans can sense. This implies that, much like mammals which have a single heterodimeric SR pair [40], these species should not be able to differentiate different sugars. We infer then that moths, through duplication of one of their two ancestral SRs into four genes, probably do have the ability to discriminate different sugars, most likely by combining one of these four proteins with the single BmGr4/HvCr5 protein in different gustatory sensory neurons. Finally, although all existing fly and Tribolium SRs each appear to have evolved from a single SR gene, as noted in the results this does not imply that ancestral flies and beetles had a single SR, because additional genes could have been lost. Today, however, flies apparently employ combinations of their SRs allowing recognition and discrimination of diverse sugars. Dahanukar et al. [9] infer that DmGr5a and DmGr64a are crucial to sugar perception because a double mutant removing both of them is incapable of recognizing any sugars. Since Gr5a and Gr64a are the most widely expressed of the SRs, with Gr61a and Gr64b-f apparently being expressed in limited sets of neurons overlapping with Gr5a and Gr64a [9,10,39], a simple model is that functional heterodimers require either Gr5a or Gr64a. An obvious problem with this simple model is that Gr5a has been lost independently from both the *D. pseudoobscura/persimilis *and *D. grimshawi *lineages, and it seems unlikely that these species would have lost such a major portion of their sugar-sensing abilities. Gr5a and Gr64a nevertheless do represent the two major SR fly lineages after an initial duplication (Figure 3), so it appears that one daughter gene from each of these two lineages has specialized in being the more widely expressed partner, while the others, Gr61a and Gr64b-f, might be involved in recognition of particular suites of sugars. It is not obvious from the Tribolium SRs which protein(s) might be the widely expressed heterodimeric partner(s) of the others.

An unusual aspect of these SRs is the origin of a novel exon from within an intron in the expanded lineage of moth SRs. Novel exons are known to have evolved from intronic sequences in various vertebrates, in a process called "exonization". Most such instances have resulted from the evolution of splice sites involving a short retrotransposon or SINE, such as Alu elements in humans (reviewed by [41]), however no such examples appear to have been published from an insect. Exonization is thought to occur with such a retroelement inserted in the opposite orientation to transcription with the inverse "poly-A" tail of the retroelement forming a pseudo 3' splice acceptor site, along with de novo formation of a 5' splice donor site within the retroelement. SINEs are widespread in *B. mori *[25,26] and likely other moth genomes, so perhaps such exonization events will be relatively common in moths. This particular event is too old for any vestiges of the potentially originating retroelement to remain. The novel exon in the four BmGr5-8 genes is short, encoding just 15–20 amino acids. The exon exhibits no sequence conservation among the four genes. These extra amino acids nevertheless more than double the length of the third extracellular loop in these four moth SRs relative to all the other SRs, and most other Grs. The origin of the one or two N-terminal exons in the Drosophila Gr5a/64e/f lineage and mosquito relatives, and hence the existence of introns a and b, is also a novelty in the SR subfamily and Gr family, but whether these evolved by insertion of introns into an extended 5' exon, extension of the start of translation into a 5' UTR exon, or true exonization is unclear.

## Conclusion

Our investigation reveals that the repertoire of extant insect sugar receptors can be traced to one or two ancestral genes in each major insect order. We are unable to say much about the even older evolutionary history of the insect SRs because the body louse *Pediculus humanus*, representing a more basal insect lineage in the Exopterygota as compared with the endopterygote insects herein, does not have SRs (HMR unpublished results). The long branch leading to the SRs from the rest of the Gr family [5], suggests that the louse should have SRs but may have lost them during evolution of its obligate ectoparasitic lifestyle. The imminent availability of genome sequences for two other exopterygote insect lineages, the pea aphid *Acyrthosiphon pisum *and the kissing bug *Rhodnius prolixus*, as well as other arthropod genomes, will hopefully further illuminate the origin of the insect sugar receptors from within the Gr family. We predict, however, that those with SRs will always have at least two proteins forming a heterodimer capable of detecting diverse sugars, as represented today by the two SRs in bees and wasps.

## Authors' contributions

LBK annotated mosquito Grs (*Culex*; *Aedes *already published), analyzed data, designed and edited figures, helped draft and revise manuscript. HMR annotated Grs, analyzed data, drafted manuscript and designed figures.

## Supplementary Material

Additional file 1 **Amino acid sequences of SRs**. We have provided the amino acid sequences of the complete set of sugar receptors used in our analyses. Sequences are presented in FASTA format, but have been converted to a PDF for simplified access. Click here for file
