# Supplementary material for: Evolution of the sugar receptors in insects
Source: BMC Evol Biol. 2009 Feb 18;9:41. doi: 10.1186/1471-2148-9-41 (PMC2667405; doi:10.1186/1471-2148-9-41)
Supplement: Additional file 1 — Amino acid sequences of SRs. We have provided the amino acid sequences of the complete set of sugar receptors used in our analyses. Sequences are presented in FASTA format, but have been converted to a PDF for simplified access. [file 1471-2148-9-41-S1.pdf]

>DmGr5a
MRQLKGRNRCNRAVRHLKVQGKMWLKNLKSGLEQIRESQVRGTRKNFLHDGSFHEAVAPVLAVAQCFCLMPVCGISAPTY
RGLSFNRRSWRFWYSSLYLCSTSVDLAFSIRRVAHSVLDVRSVEPIVFHVSILIASWQFLNLAQLWPGLMRHWAAVERRL
PGYTCCLQRARPARRLKLVAFVLLVVSLMEHLLSIISVVYYDFCPRRSDPVESYLLGASAQLFEVFPYSNWLAWLGKIQN
VLLTFGWSYMDIFLMMLGMGLSEMLARLNRSLEQQVRQPMPEAYWTWSRTLYRSIVELIREVDDAVSGIMLISFGSNLYF
ICLQLLKSINTMPSSAHAVYFYFSLLFLLSRSTAVLLFVSAINDQAREPLRLLRLVPLKGYHPEVFRFAAELASDQVALT
GLKFFNVTRKLFLAMAGTVATYELVLIQFHEDKKTWDCSPFNLD
>DmGr64e
MARTTGDPAKRRRCMSRIKFWRRSRVGSEATLGIIKYRVVEKDTKRFKLSLIKAWLLRIRQEDYKYSGSFQEAIKPVLII
AQIFALMPVRKVSSKFAEDLTFTWFSVRSYYALVTILFFGVSSGYMVAFVTSVSFNFDSVETLVFYLSIFLISLSFFQLA
RKWPEIAQSWQLVEAKLPPLKLPKERRSLAQHINMITIVATTCSLVEHIMSMLSMGYYVNSCPRWPDRPIDSFLYLSFSS
VFYFVDYTRFLGIVGKVVNVLSTFAWNFNDIFVMAVSVALAARFRQLNDYMMREARLPTTVDYWMQCRINFRNLCKLCEE
VDDAISTITLLCFSNNLYFICGKILKSMQAKPSIWHALYFWFSLVYLLGRTLILSLYSSSINDESKRPLVIFRLVPREYW
CDELKRFSEEVQMDNVALTGMKFFRLTRGVVISVAGTIVTYELILLQFNGEEKVPGCFEN
>DpGr64eP
MARTTGDPVKRQKCIARIKFWRRSRVGSDITLGILKYKVVSNQAQRFQFSKINAFLRRAVRDDYRYSGSFQEAIKPVLII
AQIFALMPVRGIGSKLAEDLTFAWSSARTYYALAMMISFGVTSGYIVAFMTNISFDFDSVETMVFYGSIFLISMSFLQLA
TRWPAIAQEWQAVETKLPPLRLGKERRSLAHHIKMITLVATTCSLVEHLLSMTSTMTYSVACPRWPGHPVDNFLYFNFAT
VFHFVDYSTFLGLLGKVINVLSTFAWNFNDIFVMAVSVALASRFRHLNDYMQREARSATTVGLLDAVQSQFRNLCKLCQV
VDDGISTITLLCFSNNLYFICGKILKSMQTKPSASHTMYFWFSLTYLLGRTLVLSLYSSSINDESKRPLRIFRMVPREYW
CDELKRFSEEVHMDTVALTGMKFFRLTRGVVISVAGTIVTYELILLQFNKEETTAFTCENA
>DmGr64f
MKILPKLERKLRRLKKRVTRTSLFRKLDLVHERARKKAFQESCETYKNQIENEYEIRNSLPKLSRSDKEAFLSDGSFHQA
VGRVLLVAEFFAMMPVKGVTGKHPSDLSFSWRNIRTCFSLLFIASSLANFGLSLFKVLNNPISFNSIKPIIFRGSVLLVL
IVALNLARQWPQLMMYWHTVEKDLPQYKTQLTKWKMGHTISMVMLLGMMLSFAEHILSMVSAINYASFCNRTADPIQNYF
LRTNDEIFFVTSYSTTLALWGKFQNVFSTFIWNYMDLFVMIVSIGLASKFRQLNDDLRNFKGMNMAPSYWSERRIQYRNI
CILCDKMDDAISLITMVSFSNNLYFICVQLLRSLNTMPSVAHAVYFYFSLIFLIGRTLAVSLYSSSVHDESRLTLRYLRC
VPKESWCPEVKRFTEEVISDEVALTGMKFFHLTRKLVLSVAGTIVTYELVLIQFHEDNDLWDCDQSYYS
>DpGr64f
MKFLPAKLERKFRRLKKHSRSSLTRKLDVMHESARKKVIEENCDAYKNQKQSEYECRKRPTKFPGGTRETFLSEGSFHQA
VGRVLLVAEFFAMMPVKGVTAKHPGDLSFSWRNVRTCFCLVFIASSLANFGLSLFKVLNNPISFNSVKPIIFRGSVLLVL
IVALRLAQQWPTLMMYWHEVEQGLPQYPSQVGKGQMGHTIRMVMLVGMMLSFAEHLLSMISAIHYARYCNSTSDPIKNYF
LRTNDEIFYVTSYSTALALWGKFQNVYSTFIWNYMDMFVMIVSIGLAAKFRQLNNDLRNFKGMHMAPSYWSERRIQYRNI
CVLCDKMDDAISLITMVSFSNNLYFICVQLLRSLNTMPSVAHAVYFYFSLIFLIGRTLAVSLYSASVHDESRLTLRYLRC
VPKDSWCPEVKRFSEEVISDEVALSGMKFFHLTRKLVLSVAGTIVTYELVLIQFHEDNDLWDCNQSYYS
>AgGr15
MGFAVSENHDTKPLIQPTWHTCLKRWCASWLHWPRVQRRASREDWLFNGTFHEASRGVLMMAQLFSIMPVCGILAKDPRK
LRFSYTAGRTFYAYFCAIGIGFLATMSVYFFASKRYHFQKMVTAFFYCYNLYAMYRFGRLGQRWPALMVKWARVDDSLPP
QKGLFERAVLAYRIKLCSIMVMALSLSEHLLSIVAAVHYSNNCPAVHDPYEAFFKSNFAFVYYYFPYSTWRGFLTKFFNV
ICNFMWSYVDLFVIVISMGLSHAFRRINAHLFLHKREKMTEQFWGEQRQKYRNVCDLVTTVDDHISAITMLSISNNLFFI
CVQILNSMNSRPTLVHTVYFWFNLIILIGRTLAVAMFAAEVNDESKRPIEVLRTIPREGWCLEAKRFAEEVTTDTVALTG
LKFFSMTRQLVLNVTGAIITYELVLIQFHKDEASDVDLCKLKRMDTL
>AaGr4
MDSRFNGELVPVQSKSRAFLKHFKYPKRATRENWIHDGSFHDAVSGLLITAQLFSIMPVCGIGQKDTTKLHFSWKSKRIF
YSYAACMGTAFLAVTSTIRFVDRNFNFSRLTGVFFYFYNLYGMYCFVRVAQKWPVLMQKWFNVEQLLPQSSNIIERGKLA
NKIKLISILVITLSLMEHMLSIVAAVYYTPNCPNIKDPVKMFFKSNFLFVFYYFEYSEIRGFVVKFINVISTFVWSYIDL

FVIIVSIGLSHTFRRINNHLMNHKREKMTEQFWGEQRQNYRNICDLVRFVDDAISIITMLSISNNLFFICASILNSLNTH
PTLVHTVYFWFGLAFLIGRTLAVSMCTAAVNDESQRPFEVLRAIPRDGWCVEAKRFAEEVINDTVALTGMKFFNMTRKLV
LKVTGSIITYELVLIQFHQDETADYDLCTFRRT
>CpGr4
MARKLVQDRLFRRRTNKVTRFNNDVAPSVERKSHFVTELNLFKRASREDWIRDGTFHDAVGGLLATAQLFAVMPVCDVTA
KDPRRLHFSWFSKRALYTYVGLAGTAFLAMNAIIRFLMKNFNFNRLTTVFFYSYNLYGMYRFLLLARKWPKLMQSWYDAE
QTLPQLGNVVDRGALAWKIKMISLLVITMSLTEHLISIIAAVYYTSDCPDIGDPVDFFFKSNFVFIFHYFEYSKVRGFFI
KYINILCTFLWSYIDLFVIVTSIGLSHTLKRINEYLMKHKRESMTEKFWGEQRQNYRNICDLIQKVDDVVSIITMLSISN
NLFFICVSILQSLNSHPTFVHTVYFWLGLIFLIGRTLAVSMYAAEVNDESKRPIEVLRTIPRDGWCLEAKRFAEEVVNDT
VALTGMKFFNMTRKLVLKVTGSIITYELVLIQFHQDEPVEVDLCKMRSF
>AgGr16
MGLQKIYPAEKSNNSTEMLSKTVRILDKDFYHPPNRPRFINGGGYFCRSIRPVLIVGQMFGLLPLDGVWCGRWWSIHWRL
LSWRNLYALFVQLGALIMACFSFATFWYSGVEFAKIMSWWFFTLNLLISINFAVLARSWPQLMSRWVQLEQSLPDQPRLS
AACRRNARQVGLVATVLLTSGLIEHVLSKPAGLHRAYRCPIPNLLEAHYKQAFPEMFSFVPYNPYIGFLAQTITSLLTVY
WNYVDLFLISVSVGLRTNLAQVNDVIASSEKLYHRGIFWKDQCTHYRRVLGLIRHVNNHIGVFIVISYASNLFFICVQLV
NVFQQNSSFIVTSYFWYSLFHLIGRIVAVSLYGSAIHDEYCRTRTLFYNLPDEYGYTDEVQRFHRQVEHDSVALNGYGFF
YLTRKLILKIAATVVTYELVLTQVNEAEAKNGDDNPCT
>AaGr5
MSKTREYRMMFFTLESVTITLTVYQKIIALWEFYRQRQVNLDQEGSYLAVFRPVIILGQVFAIFPVVGYGVALAERIQFK
WCSLRMLYTMMFQLGGAIMSGFSLATFWTTGVEFSKILSWMFFTINLSITIGFTVLARRWSAMMTEWENTEQSLPFRPQL
TASNRRVRRKVITIMVTLMLSALFEHALAKPSGLYRAYKCGIKDLLEAHLMQAFPEMFSFIPYDIYVGFVAQVVTSVLTF
YWNYVDLFLIVLSIGLRQSVRHVNEIILNSKAQYHSDTFWHDYRKHHQRVCNLVHIVGQNVAYLVVISFANNMFFICIQL
IGVLKPYPGIIVAIYVWYSLAHLMTRMVMVAVYAAAIHDESRRILPTFRTLPTQYYSKEVQRFHQQMENETVALSGFRFF
HLTRKLILKISGTIVTYELVLLQVNDAEEKNGDQNPCT
>CpGr5
MFTTSPRSDNLILVRPKGASLEKEFYQQRRTVKFDQEGSFLAVVRPVIILGQVFGIFPVVGYGAAQADRIRFEVCSLRMF
YSVLLQLGGATMSGFSLATFWTTGVEFSKILSWMFFTINLLITTSFTVLARRWPELMKEWENTEQSLPDQPLLAASNKKL
HRKVVTTMSVLMVSALFEHALAKPAGLYRAYRCGIQDLLEAHLMQAFPEMFSFIAYDIYVGFVAQVVTTILTFYWNFVDL
FLITLSMGLRQNLVHMNQIIMSSRGQFHSELFWYDHWKHFQKVCELVHLFKRKVAYLVILSFTNNLFFICIQLIGVLKPV
PGLIVAIYVWYSLAHLMTRMIMVAMYAASIHDESRKLLPMFRTMPTQFYNKEIQRFHQQMENDTVALSGYGFFHLTRKLI
LKVAGTVVTYELVLLQVNDSEEKNGDQNPCT

>DmGr61a
MSRTSDDIRKHLKVRRQKQRAILAMRWRCAQGGLEFEQLDTFYGAIRPYLCVAQFFGIMPLSNIRSRDPQDVKFKVRSIG
LAVTGLFLLLGGMKTLVGANILFTEGLNAKNIVGLVFLIVGMVNWLNFVGFARSWSHIMLPWSSVDILMLFPPYKRGKRS
LRSKVNVLALSVVVLAVGDHMLYYASGYCSYSMHILQCHTNHSRITFGLYLEKEFSDIMFIMPFNIFSMCYGFWLNGAFT
FLWNFMDIFIVMTSIGLAQRFQQFAARVGALEGRHVPEALWYDIRRDHIRLCELASLVEASMSNIVFVSCANNVYVICNQ
ALAIFTKLRHPINYVYFWYSLIFLLARTSLVFMTASKIHDASLLPLRSLYLVPSDGWTQEVQRFADQLTSEFVGLSGYRL
FCLTRKSLFGMLATLVTYELMLLQIDAKSHKGLRCA
>DpGr61a
MSKAPDSILRRLKVRRQKQRTILAMRWRCAKGGKEFKELDTFYRAIRPYLCVAQLFGIMPLSNVLSRDPQDVKFRLRSVG
MCFTGLFLLLGGIKTVMQANILFRTGLNAKNMMNLVFLIVGIVNWLNFTGFARSWSKLILPWSSLDILMQFAPYAPSKHS
LRSKLRLIGCVVGSLAVVDHLLYYASGYYSYHMHIFHCHTNHSRLSFGSYLEKEFSETFELLPYNMFSVCYGFWLNAAFT
FLWNFMDIFIVLTSIGLAQRFRQFADRVLALQGRQVPDTLWYDIRRDHIRLCELASLVDESMSNIVLMSCANNVYVICNQ
ALAIFTKLRHPINYVYFWYSLLFLLSRTSLVFMSASKIHDASLLPLRTLYLVPSTHWTEEVQRFVSQLTSEFVGLSGYRL
FYLTRKSLFGMMATLVTYELMLLQMDAKSHKAGLPDLCA
>DmGr64a

MKGPNLNFRKTPSKDNGVKQVESLARPETPPPKFVEDSNLEFNVLASEKLPNYTNLDLFHRAVFPFMFLAQCVAIMPLVG
IRESNPRRVRFAYKSIPMFVTLIFMIATSILFLSMFTHLLKIGITAKNFVGLVFFGCVLSAYVVFIRLAKKWPAVVRIWT
RTEIPFTKPPYEIPKRNLSRRVQLAALAIIGLSLGEHALYQVSAILSYTRRIQMCANITTVPSFNNYMQTNYDYVFQLLP
YSPIIAVLILLINGACTFVWNYMDLFIMMISKGLSYRFEQITTRIRKLEHEEVCESVFIQIREHYVKMCELLEFVDSAMS
SLILLSCVNNLYFVCYQLLNVFNKLRWPINYIYFWYSLLYLIGRTAFVFLTAADINEESKRGLGVLRRVSSRSWCVEVER
LIFQMTTQTVALSGKKFYFLTRRLLFGMAGTIVTYELVLLQFDEPNRRKGLQPLCA
>DpGr64a
MEGPALNARKTGPKRRHESLLRTLAQPANTVPKLADKTHLEFNVITSEKLPEYARLDIFHRAVYPFMFLAQCFAVMPLTG
IREPNPRRVRFTYKSLPMLVTLTFIAAALMMELAMLKHLLQIGINAKNFVGLVFFGCVLLACVVFIRLARRWPPLIRYWT
RTELVFTRAPYEMPKRNLYRRVQLAGMMIIGLSLGEHAMYQVSAILSYKRRVNLCSAAANITAVTSFEDYITLNYDYVFQ
WLPYSPIIASLILLINGACTFVWNYMDLFIMMVSKGLAYRFEQITARIRHLEHEEVAESTFIEIREHYVKMCELLEYVDS
SMSSLILLSCVNNLYFVCYQLLNVFNKLRWPINYVYFWYSLLYLIGRTAFVFLTAADINEESKRGLGVLRRVSSKSWCVE
VERLIFQMTTQTVALSGKKFYFLTRRLLFGMAGTIVTYELVLLQFDEPNRRKGLLPLCA
>AgGr20
MIEVGFGFRMVPGPVRSSNATRPTRSRWHWLRKRGNVTFVRPAAEIDDRKERFYLAIASVLRWARLFGVFPLSNITATDP
SAFRFHYFSPYIVLSALSIVGGLFIMAAALVRLNRVGINAMNIAEPIFFGMCTLLQLLFVRLAQAWRGFMVYWAEREEMF
FARPYGAINLRRKVIGLAVCILTSALVEHVVYVINQAYNVYQESLTCQYNVTNPLKLYGTLTFGSVYQSVPYHHLLTMYL
LYTTISLTFIWTFTDLFIMLVATGIACRFGQLNKRIDSNLQNGSEAFWGEMRTHFVGLIELVERTNRIVGPLLIASCAND
MYFLCLQTLNALEDKPYDINDWYFRYSFTFLILRTSVKLWFAADVDENSVRTHKLVQKIRSEHYNDELEILRICSSGGVS
ISGMGFFTITRRIFLTMAGSILTYELVLMRFHRSSKGVGEDLPCGYID
>AaGr6
MIDPLWRDRYILWKNSKILQPDEEPVNNEENVSEYDFFHIAIAPVLRFSQLFGVFPLNSVMNRLPGNMLYKSVSFATALS
MMAIFGGYAVSLLSLKRLARTGLDAINMAEPFFFAVCATSAVLFWALAKEWQFVVTVWSETERVFLRKPFRGKALRSSIR
RTAFVVLTLALAEHIFSVANNIANLRREVHHCNWTISSPVKYFCLKTFSSTFDSIPYNLPVALYNEYVVVAMTFAWNFVD
LFIVLVSIGLTTRFTQLNIRISERIQSRKGTTEDFWEQIRIQYVSLCDLVVLLNRSINRLVFVSYANDLYFICLQIMHAT
LEQPFLINRVYFFYSFSFLLLRTFLMFWYSSQVQDASHQPCRLILRVPNHEYCDELQRVQMYSRRGVSLTGMGVFLVSRR
IFLTIAGTIITYELVLLSFRKRIMDEPDDNNDVSCEPLHLD
>CpGr6
MNYDPYWRNPNILWKKSSILAAEPFTVAEYDKFHNEATGSDHFHTAIAPVLTMSQLFGVFPLQSVRNRAPESMTFRTVSV
TCVVSAISIFGGYAISLLSLIRQGRALNAINIAEPFFFSICATSSVIFWQMAKQWQAIIVTWSRTERKFLHHPFRKTVLK
WKIRSVATVLLLLAFAEHLLSVANNVSNLRREVDYCNWTIRDPVKFFCVRTFSTAFHTVAYSLPVAAYNEYIIISMTFVW
NFVDLFIMLVSIGISARFDQLRDRVFERMNSRTPTTEHFWEQIRILYVALCELAGTINRAISKLVFVSYANDTYFICLQI
MNASQEQPSAVNKVYFCYSFVYLLLRTFLMFWYSSEVQHASHATYRLILRVPNEEYCDELQRLQMYSKCGASLNGMGVFF
VSRRILLTLTGTIITYELVMLSYRKDSSEQHSSKLSCDPW
>CpGr7
MHTYPYWRNPNVLWKKSSILAAKPFTVVECGKPQKGASESDHFHTAIAPVLTLSQLFGVFPLQSVLNRAPESMTFRTVSV
TCVVSAISIFGGYTMLLLSLVRLGRDLNAINVAEPIFFGICATSLVIFWQLAKQWQAIAVAWSRTERTFVRYKSNLKGKI
RFVAAALLLMALAEHLLFVANNVSYMLKEIDYCNWTIPDPAKFFFAKTFTTTFHTFAYSLPIAIYNEYIIISMTFVWNFI
DLFIMLISIGIAARFDQLRDRVFDRINSWTPTTEHFWEQIRIQYVALCELAASINRAISKLVFVSYANDTYYIVLQIMNA
NQEQPSVVNKVYFWYSFAYLLLRTFLMFWYSAQVEHTSHGMCRLIQLVPTAEYCDELQRLQMYTKCGASLNGMGVFSVSR
RIVLTLTGTIITYELVLLSYREGSSDQHSTTVSSCEPWQLT
>AgGr21
MCVRSNPDKENRAPVLESVRTLATSMCSSVTTTAMKLAAKGYTLRQRVRQKLCPKIVDDDRDTFLRGIRPVVILGQMFGI
FPIYGVTRNDPKRFRLKWFSLRVILNLTVVVTALLQAYYEYGRLKAIGINAKNVSSLIFFIDACLINVLFLNLATKWRSV
AMKWDEVDDTFNRPPYHMQSWSLRKRLGVVSFTLVFLAAVEHILSIVSNVHNQMVEIKYCNWTEPNYFQHYSLRRFANIY
LNFPYNSLSAVFFTYVSSALTMYWNYQDIFIIMISIGLATRFQQINNYLKILSDGVLIPGEDFWIRVRTNYVAVCELLDD
VDRAISWTMLISCATNLYYICLQILHVSKKLANTVEDAYYGFSLGFLIVRTVIVFLSAAHIHDCAKKPLDIIMKIPNVGW
CVELERFSTQLKSEKVALSGMGFFSLTRQLLFSMAGTIVTYELVMLKFDQESESKGNIPLCTKFRRFEHVSV

>AaGr7
MQCIFIVHSFIVLTDNFQKDHYKLNSEKFYYINSVYILTGQLFGIFPLSGVFDKDPNRIRLVWPTVRVALDLIVLGAGIA
NTIAECMRLRMVGVNAKNINGLIFFVDGCIINVLFLLMATKWNRVAVKWDSVDRIFLTESYRIESKWTLKRRLWTATALL
LGLACCEHLLATINNLNDQWHEIEHCGWKENITDTFRHFSLRKFSNMYSIVPYSTVSAVFFSYVSFALTLYWNYLDVFII
LISIAIATRFDQINTHLRTLAGGGVLIPNEPFWIRVRTHYVSLCELLDEVDQAVAWIVLISCATNLYFICLQILNVSQKL
RYPMNDVYYWFSLLFLMGRTATLFLCAAHIHEAAKRPLDIVAKIPNNGWSVELDRFSSQLKSETVALSGMGFFHITRQLL
FSMAGTIVTYELVMLKFDKESEGKGYIRPCSFFDIEKKWLT
>CpGr8
MVASDKALKEQPPVENFPWSLHDTYHEAVRPLLIIGQIFGMFPVTGIFNRNPCKVHLEWISVRVVLNLVIFGAALVNAVA
EFLRLRQVGANAKNINGLVFYIDCGTISVLFLKMATSWNQVAVKWAHVERIFLEETYRVESWWTMKRKIRIVAGMLLLGA
VAEHLLSIVNLANDHRFEAINCGWQHNITDAFRHFALRKFSNIYIHFPYSTASAVFFLYVSFALTLSWNYMDIFIILLSV
AIASRFNQINAYLETLAAGGVLVPNEPFWIRVRIHYVALCELLAKVDRTMSWLMLVSCATNLYFICLQTLNVISQKHPHV
MNDLYYGYSLFFLIVRTVTMFLCAARIHEAAKKPLDIVSKIPNTGWCVELDRFSTQLKSETVALSGMGFFHITRQLLFSM
AGTIVTYELVMLKFDRESEGKGYIPPCSMFDIERQWLPSH
>AgGr19F
MNFHNTIRPILVIFLLFGQFPLYGVLTRKPLRWHFRWCSLQTVLSLGLIHVGLFLCFVEYDRLKAIGVNADNLIGPLFYL
DVIIIMLLLLRVAYRWPTVVPKWEQIESLEVMQYNARQQNARSCRRIRAIALLLIVLGFAEHMLSIGKTVNARVYEARTC
HWNYSNLPEYYALRTYGFFFRRVSYNFPSFIFLEYANTALTMAWTVQDVLLIMISDSIAGYFKRINSRIQFYTTVQVVAR
EKFWSEIHSDYVMVCELLEHVMSICSPLLVVSCGTNLYLICYQLFHLVDRTDDFIIVTVFTYFSLFFIILRTFLTMHYCS
AVHEVARKPLKLFRRVPTSNWCSELERFYSFIRKSSIAINAMGLFRLTKKTMLTMLGAVITYELVMLHFAQTTANQGIVR
ACSPEQFLFQPKMQITTN
>AaGr13P
MPREETFNTAMRPVLLIFQLIGVFPLCGILRKDSRELRFQWFTLKALLSVVIILIGLTMSYVEYEZLDRVGGVNAQNIIG
IVFFVDTVISTSLLVILAQKWHKLAVHFDEIHRIFSISDEKIIKGIHFPIRFTAGVILTCGILQHMSSKTADVYNQYHEA
EYCGWEMKNFPYYFASRNYSFIFKHIPYNILILLIFEYAATVLTLSWSCHDLMIILTSFGITFYFRKIYEKILPFHSGVM
IASEKFWMEIRSHYVILCELVKATNGSLSLIIIHSCGKNLYLMCYELINIARKDESILSSVHHWCSLINLTIQTTMVFYS
ASMMHEIAKAPLAVCNRIPNFGWCSELERYIGQLKSDRVAYTGMGFFHLTKRSILAMAGSVVTYELVMLKFAEDTEGVGD
VVPCSNLAFSKD
>CpGr16
MDSLNNALRPVFVVYQLMGTFPVGGILQRSSVGLRFRWLSVQFLFSLTLIIVGLVMVYIEYERLERIGANANNTIGILFY
VDTVLIMALLANLARKWRPLALEWERVDQEFLAKETGEAKRSLRKAVWFTSGVMIVCGLLEHLLSKTAGIINQSHEAKFC
NWEIQSFAHYYASRQYAFIFKHIPFNIPVLAFFEYCNAALTMAWTCQDLLIILISMALSHRFRQIYAQVQPFSSGIVIAA
EKFWSDIRAQHTLLGQLVRDTNRLLAPLIIASCGTNLYLICFQLLNISRKQESLASSVNHWYALCYLIVKTNLVFYHTAM
VNETARAPLAICRRIPNIGWCLELERLVDQLRNERVSLSGMGFFHLTKRTMLAMAGTVVTYELVMLKFAEDTEGIGDVKP
CSRLAFSKDT
>CpGr17
MNLNRFACYFIFSIKTFRMSQSCLKLSFMPRKSRSPSSAMDSLNNALRPVFVVFQLMGTFPVGGILQRSPIGLRFRWLSV
QFLFSLTLIGFGLLMVYIEYKRLERIGANANNTIGILVYVDTVLIIALLVNLARKWRPLALEWERVDREFLAKETGEAKK
SLRKTVWFTSGVMIVCALLDHLLSKTADIINQSNEARFCNWEIKSSAHYFASRQYAFIFNHIPFNIPVLAFFEYYTAALT
MAWTCQDLLIILTGMALSHRFRQIYAQIQPFSSGIVVAAEKFWSDIRAQHSLLGQLVHDTNRLLAPLIIASSGTNLYLIC
FQLLNISKKQESLASSIHRWFALSYLIVRTTLVFYHTAMVNETARAPLTVCRRIPNIGWCLELERLVDQLRNERVSLSGM
GFFHLTKRTMLAMAGTVVTYELVMLKFAKDTEGIGDVKPCSRLAFSKDT
>AaGr8P
MTEGGFHCIMRPIIVPVQFLGMFPIFGAAZPSSSALNFRWTSCRTAWSGIIVCCSLLMATAEMRRIARTGINFSNIIGTY
FFVDTAIIVCLTVLMASGWRRFLHEVEKLELIWTRWPYNLRRDNLKLNCWILTMFWMFFATVEHCLASSVQVYIQYQESL
RCNWIYSNAMKNYAHRNYAYIFNWMPYNVPVLFFVRYVTYSVTMAWTYQDILIMVISAYVLTRYRQFFWRIEVACNGTVL
PTESFWVEIREHYVIISEFLVHVDKMYSPLVLSSCCNDVFLICYLLLHSLKPHAYAISFVYFWYTLCFLIGRTLTTLWMA

AELNREKRSALRVVQRISSDGWCTELERYYLQLRAEVGALSGSRFFYLTHQTTFTIVAVIFTYELVMIKYSRMTAADVGI
PENCSAMAFSQD
>CpGr9
MFKYSSTSSSTRPVLSNNQRDTFHSIMQPIILSAQCLGLFPIVGITEKTTSALQFRWFSLRTVLSVTVMTSAFLVAIAEL
RRIARTGVNFSNIINPYFFADTAIIVGLLIRLGRRWEHLMVQVENVERILLKPCYNLGQVHLKRNCWLAAGSWLVLATTE
HFFATTSFVNDQRMESLRCNWTYTNAIENYARRNYAYVFNLPWMPYNIPVLLYLRYVTFSVTLAWTYQDVILIVVCIYMN
TRYRQFFTRIEVAASVERPPPAESFWGEIRYHYIALSDLLQSMNYELSALVINSCSNNIFLVCYILLHSLGPKQSTLSFT
YFWFAVCFFVGRTCTVMLTAAKFDRTLKRALQVVHRIPNEGWCDELSRYYHHLRIEKVSLSGKRFFYLTRRTAFGMFSVI
FTIEIVMIKYSKMARNSGVPADCSTLAFSQD

>DmGr64b
MPQGETFHRAVSNVLFISQIYGLLPVSNVRALDVADIRFRWCSPRILYSLLIGILNLSEFGAVINYVIKVTINFHTSSTL
SLYIVCLLEHLFFWRLAIQWPRIMRTWHGVEQLFLRVPYRFYGEYRIKRRIYIVFTIVMSSALVEHCLLLGNSFHLSNME
RTQCKINVTYFESIYKWERPHLYMILPYHFWMLPILEWVNQTIAYPRSFTDCFIMCIGIGLAARFHQLYRRIAAVHRKVM
PAVFWTEVREHYLALKRLVHLLDAAIAPLVLLAFGNNMSFICFQLFNSFKNIGVDFLVMLAFWYSLGFAVVRTLLTIFVA
SSINDYERKIVTALRDVPSRAWSIEVQRFSEQLGNDTTALSGSGFFYLTRSLVLAMGTTIITYELMISDVINQGSIRQKT
QYCREY
>DpGr64b
MPQGETFHRAVSKVLFISQIYGLLPVSNVRALDVEDIRYRWLSPRIFYSALIIALNICEFGAVLNYVGQVAINFHNSSTL
SLYVVCLLEHFFFWRLAIQWPSIMRSWHSVEQLFLRVPYRFYGEYRMKKRIYIVFAVVMLSALAEHCLLLTNSFHLSNME
RTQCKNNVTYFESIYRWERPHLYMILPYHFWMLPFLEWINETIAYPRSFTDCFIMCIGIGLAARFHQLYRRIAAVHRKVM
PAVFWTEVRQHYLALKRLVRLLDAAIAPLVLLAFGNNMSFICFQLFNSFKNIGVDFMVMLAFWYSLVFAVVRTLLTIFVA
SSINDFERKIVTALRDVPSRAWSIEVQRFSEHLGNDMTALSGSGFFYLTRSLVLAMGTTIITYELMISDVINQGGIRQKT
QYCREF
>DmGr64c
MQQSGQKGTRNTLQHAIGPVLVIAQFFGVLPVAGVWPSCRPERVRFRWISLSLLAALILFVFSIVDCALSSKVVFDHGLK
IYTIGSLSFSVICIFCFGVFLLLSRRWPYIIRRTAECEQIFLEPEYDCSYGRGYSSRLRLWGVCMLVAALCEHSTYVGSA
LYNNHLAIVECKLDANFWQNYFQRERQQLFLIMHFTAWWIPFIEWTTLSMTFVWNFVDIFLILICRGMQMRFQQMHWRIR
QHVRQQMPNEFWQRIRCDLLDLSDLLGIYDKELSGLIVLSCAHNMYFVCVQIYHSFQSKGNYADELYFWFCLSYVIIRVL
NMMFAASSIPQEAKEISYTLYEIPTEFWCVELRRLNEIFLSDHFALSGKGYFLLTRRLIFAMAATLMVYELVLINQMAGS
EVQKSFCEGGVGSSKSIFS
>DpGr64c
MKQVATRNTLQHAIGPLMVVAQFFGVLPVSGIWPSSPAEKVSFRWFSLSFLAAVGIFVFSIMDCVLSSKVVLDHGLKIYT
IGSLSFSVICIFCFGVFLQVSRRWPHLIQRTAECEQIFMQPGYECCFGRRFSRRLRLWGVILFVAALCEHCTYVASALYN
NHLQIVECKLNVNFWLNYFQRERQQLFLVLQFSSWWIPFIEWTTVSMTFVWNFVDIFLILNFRGLQMRFQQMHWRIRQNA
FQRMPNEFWQTVRGDFLDLNDLLSIYDKELSGLIVLSCAHNMYFVCVQIYHSFQSKGNYADELYFWFCLFYVILRVLNMM
FAASSIPQEAKAISDTLYEIPTEFWSVELRRLNEVLISENFALSGKGYFFLTRRLIFAMVGTLMVYELVLINQMAGTVSQ
KSFCDGGVGSSKSVFA
>DmGr64d
MLRSHLSVHGLQMERSVQENTLHYTIGHVLIIARIFGVLPLAGINPNGKPENVRFRWFSPYILFFVVAFTFVIADFMLST
KIVLNDGLQLYTMGSLSFSVICIFCFGSFIKLSRRWPHIIRETALCERIFLKPCYANQEGLNFTRFLRRWALILLVAALC
EHLTYVGSAAWSNYVQIRDCNLKVGFVENYFLRERQELFSVFEYRAWMVFFIEWNTMAMTFVWNFGDIFLFLMCRGLKIR
FQQLHWRIRQNLGKPMAKEFWQEIRSDFLDLDSLLKLYDKELSGLILVCCAHNMYFICVQVYHSFQVKGAFMDELYFWFC
LLYVISRLMNMMLAASSIPQEIKDISNTLYEVRSSPWCDELGRLSEMLRNETFALSGMGYFYVTRRLIFAMAGALMGYEL
VLFRQMQGAVVQKSICSRGPGSSMSIFFS
>DpGr64d
MEWSAQSVPNKNTLHHAIGYVLVVAQFFGVLPLSGVEPSVPVASVRFRWFSPLNLLPVAALCFVLLDFVLSAKLVIQNGL
KLYTIGSLSFSVICIFCFGAFLLLAPRWPHIIRRTFECERIFLQSCYNSSIGRRFSQRLRRWAIALLVTALCEHLSYVVS

AVWSNWKQIRECHLDIDFWQNYFLRERQELFSILPYSTWFALYVEWCTLSMTFVWNFVDIFLILVCRSMQMRFQQLHWRI
RQHIGRRMSDEFWQEVRYDLLDLNDLLKLYDKELSGLVLVACANNMYFICVQIYHSFQVKGAVLDEVYFWFCLLYVVSRI
VNMVLAASSIPQEAKQINFTLDEVPTSCWSKELERLSEIFHNEAFALSGKGYFVLNRRLLFTMAATLMVYELVLINQMEG
EEVQRSICNRGAGSSMSIFFS
>AgGr14
MLNMTPPEVTPVKRFPGVSRPPSTPSTALAEETPPADGAAERECSTHEAVAAVIFMGQLFSLIPIDGYARSTDPRDVRMR
LRSVQFVYGCVTLFIMLTLIIMLCVHTAHEPSFGVQQATSLVYYAIIVFFMVELMLLARNWSQIMGRWYTDEAPFRTDPY
RPPSRTLPFRRKVHLIAFGVMFLAFVEDTLNFVSAYRLNELHIRYCPHTAGFWKNFFHREHPYVLRVIPYHPVVGWTIEL
TMRIAKFTWHYVDVFIICLSLGLQRRFVQFNERLERLDGQPQSQGVWRALRLDYVRLSELVTFVDERFSKLILFCCANDM
FFITVQLFNSFDLKPTTVTTVYFWYSLGFLIGRCFLMLFVVSSISRASEKPLETLRRFPSTNWNLDLRRLCDAVATSENA
LSGKRFFFVRRPLILAMAGTIITYELVLLDQVKKTPDTTRDCNF
>AaGr11
MISIIHKQGYFANPLPTNLFTNRFIVTLLAVLDSYIFQLFQKMISFQTPSAPTKKTIIVQEVNVKFKAKAKGCTAHEALA
PIILVGQLFSLMPISGYFRTPISKLKFTLKSVHFAYGCFTVFIMGAIMSMFFAFRIQRGTFGIGATTTCIYYAVIITAMI
EFIILARNWPLIMQRWTADEDVFLSNPYETGQYLPLESLVKRVAFTIIFFAFVEDTINFISAYLLNVVHMKYCTHATDFW
RNFFRREHAYIVRFIPYHPALGVAIEVVMRVAKFTWHYIDVFIICVSLVLQRRFQQYNDRIRTFNGNQQPEEVWRTLRLD
FLRLSELVTYLDTKLSRIILLSCASDMFFISVQLYNIFDPKQTTVTTFYYWYSLLFLICRCFVMLYVTSSIYEASLKPLE
LLRDFATSSWNLDIQRLLDHASLKSIAFSGKRFFFITRPLILAMAGTIITYELVLLDQVAKEQDTTRDCDF
>AaGr12P
MISIIHKQVYFANPLPTYLFTNRSIVTLLAVLDSSIFQLFQKMISFQTPSPPKKKTIIVQEVNVKFKAKGKGCTAHEALA
PIILVGQLFSLMPISGYFRTPISKLKFTLKSVHFAYGCFTVFIMGAIMFMFFAFRIQRGTFGIGATTTCIYYAVIITAMI
EFIILARNWPLIMQRWTADEGVFLSNPYETGQYLPLESLVKRVAFTIIFFAFVEDTINFISAYLLNVVHMKYCTHATDFW
RNFFRREHAYIVRFIPYHPALGVAIEVVMRVAKFTWHYIDVFIICVSLVLQRRFQQYNDRIRTFNGNQQPEEVWRTLRLD
FLRLSELVTYLDTKLSRIILLSCASDMFFISVQLYNIFE
>CpGr15
MLFATKSAGSLKKGPILVVQEVKEESPIPNDCTAHVALAPIILFGQLFSLMPVSGYFHRTPDKLAFRVRSLRFLYSCVTL
FGIVSIVVLFLMYSIRRGVLGLSSAATFIYYTVITLALIEFMNLGRNWHWIVAYWTEQEKPFLYYPYSTRKGLKLDKLVK
CVASAVIFFAFVEDIMNFISAYKLNELHIKYCSHRDDFWRNFFHREHAHIVKVIPYHTVVGVGIELMMRVAKFTWHYIDV
FIICVCLSLEKRFGQFNSRIERFKGVDQPPEVWREIRLDYLRLSELVNFMDDRLSRIILMSCANNMFFISVQLYNIFELK
PTPMTSVYFWYSLLFLMSRCFVTLYISASIYEASLKPLELLRDFSTLNWNLDLQRLLDHISLKNIAFSGKRFFYITRPLI
LAMAGTIVTYELVLLDQVSKDQDTTKDCNF
>AgGr17
MESPAIDDFHRAVRPFLLISQLFSLFPLGGLYGRTLQDIRFRWFGPGTVYSFYFFLSGLLTLVAHIYYSLTVETLGTSEI
SNIIYYVLNLSGAIVLLAIAARWRTIMEKWRSLEENFLHPPYAERRFWSLKRVVAAIGSTMVVLAFVEDTLHVASVYYTN
LQYFKRCDNSTPFWTLFYQREHPKFFHYLPYSLPAVLLLELTHKIFLYVWTFMDLFIIFVALGLARRYEQFYRHAAQYKG
RHVMGPVWQRLRLDYGRISSLVAYMEGIMAPIIVCTTASDLYFIFYQMYNAFQFSASLISELYFKFSLAFLIFRTLVMLL
IASNIHVASLRPLDILRSVPMSCWTIDVQRFTQELLSGRNCLSGHGFFFLNRSVILAMAGTLITYELVMLKEVNPSSEKS
DFCDGVKRLY
>AaGr9
MQAPNQHCLAQLRKWHRHQFPSDNAVMDSNSKIDDFWGAVRPIIFVAQLFTLFPVQGVFGRDLQQIRFQWLHLRTLYSLT
FLVLAALIIIAQINHTVVSTANTSMITSVLYYILNFSGSVCFLIIATKWRNIMLNWKMYEEVFLHKPYLMKGRSLKFKVR
MVGGGILLLAFVEDLLHMLSCCRTIEVYIERCDNSSSFWETFYTREHSKTFDYISYSLPLALLLEFVHKVYLFVWTFMDV
FISVVSIGLATRFEQLFHRIEHLKGKMLPESFWAEIRLDYTKISNLVIYMDGVLSPMIMITSASNIFFITYQLYMSVQLD
ASSMTTFYYRFSLLFLILRTLVMLLTSSRVYVASRKPLEILRAVPMSSWTTSVQRFTNEILNIENALSGHKFFFLKRGII
LAMAGTMITYELVMLSEVRHSDNTQFCDGGHRLY
>CpGr11
MNSLPMDDFWGALKPVIFVAQIFTLFPVQGVLAKDVYGINFRWRSLRTLYSLFFVVLAFIALCAQINLTALFTVTTGQVA
GILYFGLNLTGAICFLIISRKWRAMMIRWKSQEDVYLRPPYRVYGRSLKFKIRLIGFSVIILAIIEDLLHVASSIKIHHK

YINFCNVTGTFWELYYNREHPQVFKYVSYNLPTVLLVEFTHKVYLFIWTFMDLFITLISIGLLTRFEQFYQRIEHLKGKS
KPEVFWAEVRGDYTKISSLVTYLDEILSPMILITCASDVFFITFQLYMTVRMKTTSITTIYYRFSLIFLIFRALLMLLTS
SHVYVASRKPLEILRAVPMSSWTTSVQRFINEILTIDNALSGHRFFYLKKSVILAMAGTLITYELVMLSEEKPLDSSNIC
G
>CpGr10
MISPNQDDFWGALKPVIFVSQLFSLFPVQGLFAKDASAIQFRWMSLRTLYALFFLTLAGLAICAQVNFIVGTKVSTGVVT
EQLYFTFNFCGTVCFFVIACRWKQIMMYWRKQEDIFLRPPYKPYGISLRVKIGVTGLSLIALSFAEDILHILSSMKVNQD
YIDFCNITEPFWMIFYTREHRKVFDYVPYNWPMTLLVEFTHKVYLFVWSFMDVFIALISIGLLTRFEQLYSRIEHLKGKP
MSEAFWAEVRRDYMQISNLVAYMDRVLSPMILITCASDVYFITYQLYKSIQLKLTSGTTFYYRFSLVFLIFRVLVMLLTS
SHVYVASRKPLEILRAVPMSSWTTSVQRFTNEILNIDSVLSGHKFFFLKRRIILAMAGTLITYELVMLSQVKATDSEIKC
ED
>CpGr13P
MSSLPMDDFWGALKPVIFVXQIFTFFPVQGVFAKDIYGINFRWRSLRTLYSLFFLVLGFIFLCTQINLTVLYKGTLWEVG
GILYFGLNLTGAIYFLIISRKWRSIMIRWKSKEDVFLRPPYRMYGRSLKFKIRLIGFSVIILAIIEESLHLASTIKVHHK
YINFCNVTGTFWELYYNREHTRVFKYVSYNVLTVLLVEFTHKVYLFIWSFMDLFITLISIGLLTRFEQFYQRIEHLKGKS
KPEVFWAEVRGDYTKISSLVTYLDEILSPMILITCASDVFFITFQLYMTVRMKTTSITKIYYRFSLIFLIFRALLMLLIS
SHVYVASRKPLKVLRAVPMSSWSTSVQRFINEILTIDNALSGHRFFYLKRSVILTMAGTLITYELVMLSEEKPLENSNIC
G
>AgGr18
MYEKAANHFNKTMSIENPHIRKGNGYGRGNWKSFGRQYVRPCVGSFHHAVAPVLLLGQCITLLPVVNIFSANYRTARFKL
RSFRCIYSLVYLALTGIYCTLFIRWYIRKGLNLAYFANCIYMVVVYLSAWLFFFIALRWQSILGAFSRCERSFLSDHYRR
QSKGRFALGWKIRFTGFAIFALALVEDWLNYYSAYQSNIVQIATCNRTNVTVWQNFYLREHPHVFHWLPFNGFTIAITEW
INRCMRYTWTYLDIFIISFCYGAQFRYEQIFRRLVAVQGIACPTNFWHDVRMDYVAVSELVQVLDAQFGHLILLACANDM
YFIATQLFNGFQRRRVIANYVYFWYSLLLLMFRTIVMLYVGSGVYAASTSPLQLLRNVPSQHWGIDLQRLTDEVASGENV
LSGKQFFFLKRQLILAMAGTLVTYELVLLDQVKKIPDTSTDCSYF
>AaGr10
MSPSVDMKLLTLTSRRAATAVAIGKESFFPNFHLPRWSKWIGRLKGKSTFDGSFHQAVAPVLFVGQCFTLMPVVEIFSHN
PRNTRFKLLSVRFAYTAIYLIAAGFYSILACRRFILRGLNVSSFADMFYLMFNYIITVLFLLIAIQWQKVLKEFANCERL
MLKDAYTKLTERVTRFNLAWRIRMVIAGIIVLAFFEDFLNFYSAYQDNYVQMDYCNRTEISFWENFYIRDHPQVFQYVPV
NIGSILFVEWINRCLRYTWTYLDLFIISFSYAAQFRYTQIYQRLVSVEGTHYPTTFWREIRTDYVAVSQLVAFLDEQFGH
LILLSCANDMYFIATQLFNGFQRRPAFMTMVYFWYSLALLIFRTLCMLYIGSGVHVASMSPLNILRNVPSKYWGLDLQRL
TDDVASGENTLSGKKFFYLKRQIILAMAGTLVTYELVLMDQVKQAPDPTTDCSFY
>CpGr12
MKLLALTSRHFQLPWPQPRKGSAAVSSSYHGSFHQAVAPVLFIGQCIALMPVVEIFNHNFRRARFKPLSARFLYSMLYLV
IAGVYGVCTCRWCFVKGLDVTLFGDSVFIVVVYMTAVLFLLIAPQWHTVLKMFNECEKIMLRDTYRKVTERYTRFNLAWQ
IRLIAFGLLVLAFIEDALNCNSVYKGNVVNLQFCNHSNVTFWENLFIREYPQIWRNIPVNFGLVLIIEWIIRCMRLTWTY
LDVFIISFSLAAQFRYNQIYYRLISLPSVASLPSTFWRNIRTDYLAVSQLVAFLDDKFGHLILLACANDMLFIATQLFQG
FQRRPTFATIIYYWYSLGLLIFRTLCTLYVGSGVHVASMSSLNILRSVPSEDWGLDLQRLMEDVASGDNTLSGKKFFYLR
RQIILAMAATLVTYELVLMDQIKQAPDKTKDCSYF
>CpGr14
MKLLALTSRHFQLPWPQPRKGSAAVSSSYHGSFHQAIAPVLFIGQCIALMPVVEIFNHNFRRARFKPLSARFLYSMLYLA
IAGVYGVCTCRWCFVKGLDVTLFGDSVFIVVVYMTAVLFLLIAPQWHTVLKMFNECEKVMLRDAYRKVTERYTRFNLAWQ
IRLIAFGIMLLAVIEDSLSFNSAYQGNVVNLQFCNHSNVTFWENFYIREHPQVLRNIPVNFGSVLIIEWLNKCMRLTWTY
LDVFIISFSLAAQFRYNQIYYRLISLPSVASLPSTFWRNIRTDYLAVSQLVAFLDDKFGHLILLACANDMFFIATQLFQG
FQRRPAFATMIYYWYSLSLLIFRTLCMLYVGSGIHVASMSPLTILRNVPSKNWGLDLQRFMDDVASGDNTLSGKKFFYLR
RQIILAMAGTLVTYELVLMDQIKQAPDGSKDCSYF

>TcGr4
MLNNSAQRINNFFKLILSLGCFLGFFPSFQMSFAWKSWTTFYTIFITSLSVIYNFIPILWVVNYHQQNISFEDVEIIIHS
AAVVFNFFILFGLAKNWNKIIDEWHNMDTTLNKNFSYPKHLKLLVTALTTLFLLFGIVVYFLKQMAFFSKLSETEGPISI
ELFYKHSGKTIFWIIPYNWITATIFTFLQFNSFIVFIFTDILLISISLILANRFQQLSKTLTKRQKIPQFHPDNASFWKN
VRKDYCKLSSLLFLIDDHISLAIIFSYCLNFFSLLRFLTKLLRDSEQNLIVKFHDYCDALNFTLRIICLTLFSSWINEAS
QEPVAILNSVVEREYSAEVGRLLLQIGFDEVALTGCKMFKLNKGLFLNIVSAIVTYELIVIQYNNN
>TcGr5
MVFAELWKKIRNRGTPPKFELPTTHNCLKKVLLLSQIVGVFPLNHLNEEPEKLHFTFKSWKVLYTSLTSFGYLFCASLSF
YKAFKIGILLNQLITPLFFFHSFMTSVLFSQVASRWPLFLNEWTKIEINLLKHYQSTTDLHKKIRYSAFGMIFVALLEHC
FSMLNYVYSSKCENNSTGVEHFFKKQFHYIFTYMPYNIVFGLCLTFVSWTAAFVWNFGDIFIILLSMIMTERFRQINQQI
QKKAEELISTPSPNTKNFIKKADYQFWRQIREDYDHLTNIVRYLDQILSNLVLLSYSCNLTFILIQLFNSLRQMKSAGES
IYFFYSFGFVIMRIVFVSIFGAFINEESQAALSYLTSLPTEHYNEEIQRLVTQTHIDSAALTGHNFFRLTKGLVLSVAAA
IITYELVLIQFNQATLNKYMQANETICF
>TcGr6
MKVLSNDLFHHRVKWILLLGQTFGLLPVNGVTSEKCNLTFSWYSKKVFYSKIIISGSIFMTTTSFYRILNSGYNLTTFGS
FIFNANSAIEGIIFFNLAKSWPQLIEKWSRVEMALDNWKNDKSLKRKFYLTICTIMSAAAVEHILSIVNTCSQIPSEVED
KYTTYFLNTYPHLFNFFEFSVPLAIFAVVMNLCNVFVWNFLDAFLIIISIALTEKFRQVTAKVIVAHNEKIHLKHHWVKL
REDYNQISILCKTVNKKISTLIIVSFGTNMFFIMSQLYWSLSIVKTTPVESIYFSFSFGLLVLRTIAVTLFASNINDESK
KSMNYLLSLSSDIYNSEVERFAFQIHSQPVALTGNDYFTITRGLLFSMAGSIVTYELFLIQSNEAVSY
>TcGr7
MRLIRKMKSDLVLTIEASDAVLAYPQPSFHQTFSFVVIFGQFFGIMPLHGVSRKNVQEIRLEWKSFRFVYAVYNIFGAFV
MGLFCILKFALDGLMLDKATMSFYVLNFFGSIQFIIISKHWVTIMKEWSFMEMSMRNYGSSINMKKRFVVMTSVIMTLAL
VEHLLFIANAFITSQSCENATLYSGDEMYFRVAFPSVFTLIDYSLWKACFVEIANILSTATWNYTDLFIILISCSLASRF
AQINHRLKNNKLLHEKFWREIREDYNKLAHLTAVVDRNIAALVVISFVSNAFFICVQLYNSLKIRVGTVETVYYFFSFGF
LVARTIAVTLYGAWINDESRKPLEILHSVPSEHYCEEISRFIQQINSSPVGITGSKFFILTRNFLLKMASTIVTFELMFL
QFGPLINTNINHRSTDCFM
>TcGr8
MTTTTHSSLRFILIAAQIFGMFPVSGVAKKDPTFLKFKWTSKRTIYSIIFALAAVVNTIIFLVHRVSLGRLKFADWVTLV
FFSVTFLIVVLLLQIAKHWPSLMKKWTQVDEAMSGYGFPPKLERKLRIIFAITVVASLVEHGLFIGVEYMSCRGNNLSEA
LDRFLMFHYDYVFALVPYHVVLGIILEIVNIFSTISWTFMDLFIILVSLSLSARFKQVAKYIKFLVERNVLNKNSWQRAR
QDYTRLTNLCKDLDEVMSSTILLSFGNNIFIILVHLYNSLQKPLEFGYLDEIYYLYSFICLLVRISAVALHAATINTESK
RPIYLLSTIPHHRYNLEIDRLLLYTKYETAALTGYKLFRITRTLILKITLAIVIYELVLVEYLKVESY
>TcGr9
MKENTTQNCLYFMLVFAQCFGMLPVTGISEENCKNLKFKWSSKRTFYSLTFALVATANTVFFLIKMIRGKRNEFQEWVAL
LFFFVTVITVVIFLDVAKKWPQLMKKWTEVDTAMNSYGFPIALSKKLKTTTLLVISAAIVEHGLFVAVASAPCEGKNWSE
LFNNYFKMRFDYVFDVIPYHFFWGIMFQVLNIFSTVSWNFMDLFIILISLCLSERFKQVATRTNQLAAKEVTDERIWEQL
REDYTRLTILSHTVDKTLANTVVLSFANNLFVILVQLFYSLQAPPRFGILNKLYYVYSFAFLIIRMVAVALNAATINNES
LKPKYCLNTLPHFLWNVEIDRFIYQIKYSPVVLTGHKLFKITNALVLQITVAIVTYELIMIQYVRVKWS
>TcGr10
MRVQPSNTPPEKSQLSTADTANLINKSLGKHNLQKSFAKVVILAQIFGFFPAQGILGRDFRAIHFTWASARVGYTIVTIL
GATFVTVLQLHKIFAKGLNVIEANRLFFYCSGLASGYLYLKLAMKWPRFMKDWSCVEVMMASYGWPAGLNRRLNVLLAVF
MSLALIEYILMQTNKLVLALECNNSTSEGFDYFFGKMSYSHIFSLMDYNIVMALVLQFITLQHTFIWVFNDVFVMLLSTA
LAYRFTQVTDRTQSMSESKNKSESAWKNLREDYNRLCRLCKRVDEEISYIVLMSFASDLLFILIQLFNSLRQMKNNLERI
YFYWSFGFLIVRTVCLCLFGGKVNDESTQPMLVLNSVSADVYNLEIQRFIHQIGTLEVAFTGKNFFSITRGLILSIAGAI
VSYELVLMQFNDSLLETISEQIDSCPVYL
>TcGr11
MFKSPTNMEKSTKIFPVPGDKNLHKSYSKMLILAQIFGFFPVQGVRGPDFRSLRFSWKSARVVYALFTLLGTFLISGFQM
QKIATKGLDLLEANRLFFFLTGVMASLLFLNLAKRWPKFVKDWCVVDATFASYGWPKGLNKKLNTLTVVFMLIALVEHIL

VQTNKLVLALECNNTTAEGFTYFLGNMSFAHIFALVEYDIFKALVLQTINLQITFIWTYNDLFVMLISTALAYRFGQITR
RIAAVASEKIKNEIIWKKLREDYTRQCRLVRKVDKEIAYIVLLTFASDLLFILIQLFNSLRRMRNDLERLYFYWSFALLI
TRIVCLCLFGAKVHDESIKPLLTLNSVPTEIYNLEIQRFIQQIGNSDIAITGKNFFSITRGLILSIAGAIVTYELVLIQF
NVNLLQGELNKKGSCPVYV
>TcGr12
MKKNSKYVQNCIFYAMKRPLLVAQIFGYFPLYGTNSDPTCLKFKWISFKTTYSVFTLFVTFFIAVCQLHKMIAVEMNILQ
MNYFVFLLCSILVNIAFIKLATEWPQLMKAWLKIELLVGNLGMRRNFRKKLDFIFTFITLLTIVEHLLMELSRAIDSVAC
SKTVSDGIRHYYVNVTFPHLFNGLVDYSLWKALIFQISNLQTTFGGTFGDTFIILLSMAFATRMKQSRTKIEALVKSHVK
ATTPWRKIREEQCSLLYLCTLLEQKISYLVLLSFCSNLYFVLVQLFSALKQMGDTLQKTYFFISFGILIFRIIFVSLSAA
SINEESRKILILLLSTPSELYSVEVERLTNQINYKAMAISGKNFFIITRGLILKIAGAVVTYELVLIQFNKKLLNEFDET
SVQQLEILHNNTYWLC
>TcGr13
MKNIISHNTENTIHSSLKFSLKILHIFGLFPVSGLSGPDYKSLKFSWRSFKFLYSLCFFCILCLFVLTLLYNVFFVKEAT
QEITNVLFYLSAAATNAVFLQLAKNWSRFIHEWHCVEVIMGSVAINHSLKKRLKIITIVILVVATVEHLLIQCYIAVSIF
GSSSFEADLRQFYKTAYSAIFTVIDFSLCKAILVHAITIRSTFSWTFIDVFIMLTSTAFVFRLKQLNAKVEMLKNARVKN
TALWKQLRYEHYRLYQLSVLIDNNMSYIIIVSFATNLYFIIIQLFGSMKIVKGTLKTAYYLISFALLIMRLISVCLCGAS
VHSESSKVLPLLFSVSSSSYNCEVERFIDQVIKNEIILTGKKFFKITKQLILQIAGAIVTYELVVIQFNLRLTEDNDSNE
ANLLA
>TcGr14
MLFSSAAKTRPKPKSPQHEKENVHKSLKSIIVIAQIFGYFPVQGVLSNEPIFLNFSWFSLRVVSSLVTIITGVVIVFGHI
RLMAVGGGYSQFEMNGIIFYACGTLSCIFFLKLSMEWQSIMLKWQEVDLKMSSYGWPKNLNRRINVTSAIFLLLEAAEHV
LIQSNKLVVAIQCKGSFSKGAEHFFVNMSFPIIFDLIKPYYGAWLGVFLQIVNTRMAFSWTFIDLFIILMSCALAARFRQ
INTRIRCLTNMKVTSEKSWIALNDDYNRLCHLCFLLDEKLSYIILTSFLNNFYFIIFQVFESFYLHKATTLETVYYFISL
GLLILRLVSVCFYGSWINEESKLSLDLLSYVPREVYNKEISRLVETLKFQSVGLTGKRFFKITKQLILKITSAVVTYELI
VIQFRKKMERDEKPYSRNSIC
>TcGr15
MAITVVAPQSKTKPFTQKTVYYTLNFFFVLLLFSGYLPVQGLFASPIHLKFKYFSREFFYSLYIIVVACIMHFLRLYHYF
TIKNDKITLGELIFMCTAFLSLVFFLQLARKWPRLIQKWCHLDEKFNVTYSYPKYLKLQLTLVTSIYLLIVFGEYFYFMS
VKLDGSENTFQKVVEKIYFYIFAHIPYNIAVVLVIVLPIFSNFIALSLIDVFVILVSITITFRFQQINELLKEQKNKNNS
INFWMTVRRNYSEIGRLVSEINDTISSITVLSYGSNMILLINELSSFINVVVEESKCFYVYSFLVLIIRLLSVYYFSSRI
NAESRKPIRILFDVSSEIYNVEIKRWFMQMKLDSVALTGSTFFRITPGLILSIAGAVVTYELIFIQFSQPK
>TcGr16
MQSSEDGDHIQNSLKLLIKIGKVWGFFPLINSSNEQVSFKFRSCCFIYSLFVWTVLFYILLMAVYSCCVMPNFFRYNFVH
VMYSSNLFCLFTIYLRLAKKWPDIITYWKRVDQAMEPKYGYPKNLTRKVKRIVIFWACTSVVNQIVVPFAPMYHLIVHGK
LQFPLSPFAIFMHTILFLQSIIGKTTVHYADLFIVAIAISLKRRFQQITQRIVNHKEQYKNVEFWRNIREDYDRLARLTQ
FLDKELSYLILFSIGFNCFWVMKLLYNVLRFGAESQTINFMFSFGFILLRFLSVFESCTRLNNESRKPSFVIQFSHIPVD
NIEINRLIQQINFDKVYFSGCQLFKIKNGIILSFAGALVTYELTLFQYHAFYLT
>TcGr17
MKKIRIDNSRNQRKTPRKKRPQLINALLLLMKSGKYLGIFPLKRVTSFEEKYIDFKSCVFIYSSLVWILNFVVVLLTLSD
WVYTNFSVDKFVSLVFNSEIFFSLTLFIYLANNWHFVMSFWNQVDQKMEYKYNQPRGFNRNVKLGLVVYSILSVGNHFGS
PMHSVVSLFQSQNQITEVTFLPQYNDFFSKIFTIHSILGKTTMHYRDFFLIIITLILREKFKQINQRIQDNSENISTDFW
QEIRNHYDLLSHMVRHFDKEVSYLILASVGFNGFWVLLFFYKSYTAKFSSSANLYFAYPFGYIILRFLLLFHCGALLNEE
SKKPAIIIHFSEIPVLNTEITRLLTQIEFDNVFLSGGKIFRMKSGILLSVVGATVTYELTIVQYNIFSN
>TcGr18
MKAPQSGLLLIQPQNNFHKSLRFLFVIAQVFGYFPVQGVSEEKVTGISFTWFSLRVLSSLLTAFLGIVVIFAQIRFMKLI
TGYEQAQMNAIVFYGSGTLSSFLFIKLARDWHEIMTKWNQLDKALISHGWPKGLDKTLKRIAIIFLALEAAEHFTIQANK
LIVAVRCRGSFSKGFRYFAVDMSFAVVFDFVDYAHWLGMIFQLMNTRMAFAWTFLDLFIILVSCALSERFRQINDRIQHL
TDIKSKHFKEWIAINEDYNRLCLFCDYLDDKISWLILISFFNNFYFIIFQVHLSLNLVLAPPLEIIYYLISSSLLILRMV

SVCIYANRVHEESKSPMDILSVAPLEIYNQENIRLIVTAKYQTTGLTARKMFLLTKNLIFAIAGAVLAYELIVIQLNKNQ
PRKQGPSIYSLC
>TcGr19
MILFPRHTEMDNTQKWSNSFKYVFFLAQCLALLPVNGKHQIYSKWKSLHILYSLFVIFVTSILLIFQIVFAVTHEFDRNV
LSSIMMKIYSICALFLNIRLGSRWQKIHKQWNQVDCVMDRRYGQLKKINLRIIAILSVYFATSLVNILKLQGTSNILEFE
LYSFVFNYVPPNYAMALIFLFVYFIYFFLSNFIDVFIASLSMAIALRFKQIRTRLELSEKSQFDLGMTEFWLEMRRDYDR
LSHLCKELDDGISGLILMSFAYNLFEVISYLFHQLMMDSQQNVAFYFFFPYMVLRLLAVCLYTSWINDESLAPVNILNSV
PSRNYNPEIGRWLVQMSFDNVALTGWKMFKVTRGIFLGVASIVVTYELVIMQFYGFSGKT

>HvCr1
11MGPTSLKRNMFFWIPVKKNKVDVAKPKVKNITTFQDALRATLIIGQVFSLLPFVGVFTNVASNVKFIKTSWKCGYSLL
SLIGQMFMAVLCVNKLAKSNVSLNGTSPVIFYVTTCVTMMLFFQVARRWPALVQHISKAEDMDPNFDCSLTRKCNITCAV
VLILALLEHILSLLSAFAGASACYTGMDTYQGFVTHFYPWVFNYLPYSIVLGVITQFLHFQSTFIWNFSDLFVICMSYYL
TSRLEQVNRKLLAAQGKYLPEIFWRATREDYCRVTQIVRKVDEVISGVVFISFANNLFFICLQLFNTLEDGLKGTGECTQ
LNSQSKLKKIVVSKSGPLGGHEAAAYFLFSLVYLLSRSVAVSLIASQVNSASSVPAPVLYDVPSPVYCVEVQRFLDQVNG
DKVALSGLQFFSVTRGLLLTVAGTIVTYELVMFQFNSSTPSLNITSPTSATHIITTLAT
>BmGr6X
MLLRNYKQNLSFWTSAKKSKIHKIQSQETVTFQGSLKLVLFIGQLFSLFPVCGLLSNDANKVXFVPISWKCGYSMLSMIG
QLFIIVMCILYVAHFETTLNGTTPIIFYGVTFISMIAFIRASRRWPELIQHISKSEELDPSFDFRLKKKCNITLLLVLVL
AILEHIFSIRSAYSASQICYPHTGFYEGFVRYLYPWVFDFLPYSEELGMVTQFLNIQSHFIWNFTDLFVICMSYYLTSRL
DLVNKKLLPAQGKYLPEIFWRTTRETYCRATKLVRKVDEIINGILFISFANNLFFVCVQLFNTFDDSVDMVGLCYNYSER
RTKPVGREPVIYLLFSLGFLISRSITVSLIASQVNLASTVPAPILYDVPSAVYCVEVQRFLEQVNGDNVALTGLQFFSVT
RGLLLSVAGTIVTYELVMVQFNQAPASDSFTEKLVENNISTIETFYNYS
>BmGr5N
YFAFYLSTGCNTFIFLRVASKWPTLIKHVYETQLDSYIDVKVKNKCFAAYIIFFSMSMTEHMLSLLSKFVITMDCLPKGS
DLFESYIIRNFPWLFEFDVPYYLPIGVILQFLTLVSTINWSYSDLFIVCMSIYLTSILKQINKKIEMAGNSNHLPIPFWR
TLREDYTRATRLVRSFDDTISSVIFLSFASNLFFICLQLYNILSNGVTSKYNLLKEMCPNYPSGPLGGYEQIMYLLFSLS
FLLGRSLVVSLVAAKVHSASMVPASALYNIPRNMYCSEIQRFLDQVHGDKVALSGLRFFYVTRSLVLSVAGTIVTYELVL
LQFSNED
>BmGr7
MVLEAHTQIQYCTAKANYCEFHAGLRHLMRLARWAGFFPVQGLSQTNPDDVRFEFRSLYALYHAITVIGQTVMTFLAFYS
FVDSNVSLSVVSNFLFYFTNYVTLVLLWRLSKNWSALISKTLEFEQSVTEIRTTRNLVSRTNTLTYVVLIFAMVEHALSK
VFNIRSVMCCLGETSLNHTVINNYFKFKWKFVFDYFSTSTTYSYFVGFIAEFLCMQATFLWSFTDVLIMCFSIYLSSFFE
DFNSTVSSFMKKASKTVPWSTLRVQYSQIVLIVKQMDEQLDYFVLISYFTNLFFICFQLYNSLNRIYDANDVCNENMDII
ATASVTYLTYYVFSFLFLVTRALLLSIMAANVHSCAQVPQLALYEVPTADYSLDVQRFQLQLRYTTVGLSGVCFNVTRGM
ILRVIGTIVTYELVLIQLTKKNLDNDTSIRDYYLPKHLI
>BmGr8
MAPRSVRSMVGTSKKDMLKGGFYETVRIPLYIYRLIGILPISGLWHRSSKYNRFSLKSFYTIIYAPTIVMQTFLLLVHIY
DLFAFFFGHQRLGRLIYHMNFYTITILIFMGSRKWKNVIKEIETIELTLPRLRNSKKALALTKSFVFAFFVFSLAEVVLI
LQFTLRLTKQRHVLPGDSGLYLRSYFVYIFPYLYDHFPFSYVMGFIVQIIKVQGIITLNMVNCSVVILSIYLTNRLKHYN
RIVFAKGSKTNNTRLKWVELNLLYTRISNLVKIIDKNLNPFVFISFTANLSYICAQLFYILNKLTSSRTVKITSFLEDKR
CDWETVLYISISFALVVLKVLLVSIIAAEVHTTSREPLRLLYTLPTAEYTIETQRLMTQVYYSNLSLSGLNFFHITRGML
LGMVATLLTYEIVLLQI
>HvCr5N
AYLDKVFLWSCLYGVFGSKRFISLIWSTLILGSLVIIEVLAIWKVIRALAGVARDMSGHRSVTARLAGTIFYSISILSLV
LVSKLYYNWRTNIAGVWGKVERSVGVKIPVDRTLKCRMTFVAGLMTFFSIFEHAMSILSSVGLDCPPSLILKRYVLVSHG
FIFMGQDYSEWFAMPLVIISTIATLLWNFQDQVIVLISMGLTSRYRRLNECLAKVCELEKQHKDSDKKIEAVKVYTWRKI

REAYVKQAMLVRKIDVALGGIVILSCSCNFYFICLQMFLGITQGLSSDLLSLIYYVISLAWLCTRVISVVLAASSVNTHS
KLALNHLYNYETHCYNVEVERLQDQLTKDYIALSGMGFFYLNKTILLQMAGAIVTYELVLIQFDDQGNDALNATKI
>BmGr4
MDKDKFQEFLPTMSRIFSMTRYFGVSTCKPSIAFGWTVILLLMLLAIEVGAIWKIVRLLGGWAVHSTDSRGFTARLSGCI
FYGNALLSLILSIKFVSSWEQLSERWSRTETDPGLRLPSDSRIKRRTVLVSAFVMTCACVEHMLSMMSATGFDCPPEEYT
ERYILSSHGFLVQNDEYNLWLAIPIFIMSKLATALWNFQDLIIIILISMGFTSRYNRLNTYVHRVVMLERNLKEGAQVSSE
NYMRFQIWRRIRQAYVRQAALVRLVDDQLGALVLLSNVNNLYFICLQLFLGINSKDRGSFINRLYYFISLGWLMFRACGV
VLAAADVYIHSKKALISLYLCPELAYNLEIKRLKYQLKNDEVALTGMGLFSLNRELLLEVAAAVLKYELVLVQYDK

>AmGr1N
SEPVAFSANSFNPKTDSLHASMRPIIMLAQFFSLFPVSGVNSPDSSYLRFTWRSPKFIYCTISFLSSSIMTIFNVLRIVT
TGISSIKMTTFVFNGTNLIASFLFLKLAMRWPCLMVTWEKLEKELSQRHRKISKISLSMKFKIVTIVVMTFALVEHSLSI
IHGYFKAKECIEFHREQSILGVYFQMQFPQIFSRTSYSLWKGILVDIINILSTFSWNFVDLFLILISIALTDQFRQLNSR
LYSIRGKAMPEWWWAEARSDYNHLATLTRQLDSHISIMVLLSFATDLYFICIQLLFSFNPMRGIIEKIYFGFSFGFLLAR
TTVVSLCAATIHDESLLPAPILYSVSSSSFSTEVMRFLSQVTTDNICLTGMKFFSVTRSLVLTVAGTIVTYELVLVQFNT
TQQTDASNATIVCEVK
>NvGr1
MSQLHFRSSKIDSSPKRGRYESLKMDSLPADVSRPSASNSGANLVESTRSFHCALRPIIILAQCFAVFPVSGVRSPDATH
LKFTWRSFKILYCCLSTLGSIVLMFFSVYRLATTTISSNKTSNLVFSLTAGITTLLFLKLARQWPSFAVSWENMERELAT
RHNPRRSSGINLATKFKILSVVVMVFALVEHTLSILSGYVSAVECASLRGDKDIMATYFALQFPQMFTDSNYTLWKGLIV
QFVNFLSTFSWNFMDLFLILVSVALTDQFRQLNQRLYSIRGKAMPEWWWAEARIDFNRLATMTRRVDSQISDIVLLSFST
NLYFICIQLLNSFKPMPNAIQTVYFCFSFGFLLSRTSAVSLYAATVHDESLLPAPILYSVCSASYSTEVRRFLTQVTTDN
ISLTGMKFFSITRSLILTVAGTIVTYELVLVQFNAVQAEHQQSESNITKVCEVK

>AmGr2
MHSEDQIQLMMLKTKDGLGEIPKGKGRGSNLKIWSSVMYHKDDNNIEDISANQENDLSTKRPRAERNYFRNSEALENFHC
AIGPVLKAAQIFGMFPVSGIGSSSLSKLQFKIFSLLTMYSGFIALMISFMTIVSMIHMLKTFNASTFQIRGGLGAATVGA
VFYGNSLVGSILFFSLSSRWVSLQYEWRAMERYIDSNSTEPTRLRWKFFIISTMVLVLSLIEHVLSIFNNIDGYEWNESN
STFHNFLEIYTLRSHSFIFDTLNYNFVYGLYVFVVSKLATFTWNFTDLFIMLVATGLAERYKSLNKKLAVTMTKCQAAFN
WRELREDYAILSCIVKKVDDHISPIILLSFANNVYFICLQLLNGLSISDKNSVLSEAYFFGSFAFLICRTCAVTLLTARI
HDQSKQSLPYLYNCSTSSYSVEVQRLQCQLATDDIALTGLRFFSITRNFMLAVAGAIITYEVVLLQFNGK
>NvGr2
MTAEAKPKGHRNASPRRIHFGIGRKMMSFFRSSKSGRGKPRSQHTPIFSKIHPAKRPALHSRRQSSNEDEPECFHRAIGN
ILLMSQFFGILPIRYIRSSSVRNFSFYKFAPRVIYSYFVLLAISVMTSISFLHLFRTLNANSFQTKGGIADATVGAMFYG
NSLLGNLMFLRLCPKWISIQHDWRAMERLIDNNGKWKGPVLRWRFTLISSTILSLALLEHILSMVNNTPSDVWFGKKNLE
DFLIIYTNKSHRFIVRNVDYNFTLGLFIFFISKVSTFTWNFTDLFIMLVSTGLAERYKRLNARILEATPAQLSVTDWHEL
RECYAVLSALVKKVDNEISGIILLSFTNNIYFICLQLLNGLSPSTAEHPIINSIYFFGSFIFLIGRTTAVTLLTARINDQ
CKLILPILYNCPSVNYCREAQRLQQQIATDDVALTGHRFFSITRNFMLAVAGAIVTYEVVLLQFNIALQRDEELNNMAAG
SNG
